# Supplementary material for: Hippocampal-entorhinal cognitive maps and cortical motor system represent action plans and their outcomes
Source: Nat Commun. 2025 May 3;16:4139. doi: 10.1038/s41467-025-59153-y (PMC12049502; doi:10.1038/s41467-025-59153-y)
Supplement: Supplementary file 1 — Supplementary Information File [file 41467_2025_59153_MOESM1_ESM.pdf]

SUPPLEMENTAL INFORMATION

Supplementary Figure 1	Goal-directed Action Task
Supplementary Figure 2	Behavioural performance in the two Comparison Tasks and correlation between Comparison and Rating Tasks
Supplementary Figure 3	Distribution of distances covered by each direction in the abstract action-outcome space
Supplementary Figure 4	Cross-validated representational similarity analysis (RSA) for the grid-like representation of the abstract action-outcome space
Supplementary Figure 5	Grid-like representation of the abstract action-outcome space
Supplementary Figure 6	Distance representations of the abstract action-outcome space
Supplementary Figure 7	Control analyses for the representation of individual actions
Supplementary Figure 8	Representation of individual actions in the SMA
Supplementary Figure 9	Interaction between map-like representations in the hippocampus and individual action representations in SMA
Supplementary Figure 10	Speculative schematic representation of alternative interaction models between SMA and HPC-EC System
Supplementary Figure 11	Trial structure of the first Comparison Task
Supplementary Figure 12	Reconstructed map-like representation obtained from the two Rating Tasks.
Supplementary Figure 13	Different models of pattern similarity values
Supplementary Figure 14	Effects of landmark outcomes on the behavioural performance from the first Comparison Task
Supplementary Table 1	Significant clusters of the abstract distance-based BOLD adaptation analysis of the action combinations
Supplementary Table 2	Significant clusters of the abstract distance-based BOLD adaptation analysis of the landmark outcomes of actions
Supplementary Table 3	Significant clusters of the action similarity-based BOLD adaptation analysis of the action combinations

Supplementary Table 4	Whole-brain clusters of the generalized Psychophysiological Interaction (gPPI) effect with left HPC used as a seed region
Supplementary Table 5	Whole-brain clusters of the generalized Psychophysiological Interaction (gPPI) effect with SMA used as a seed region

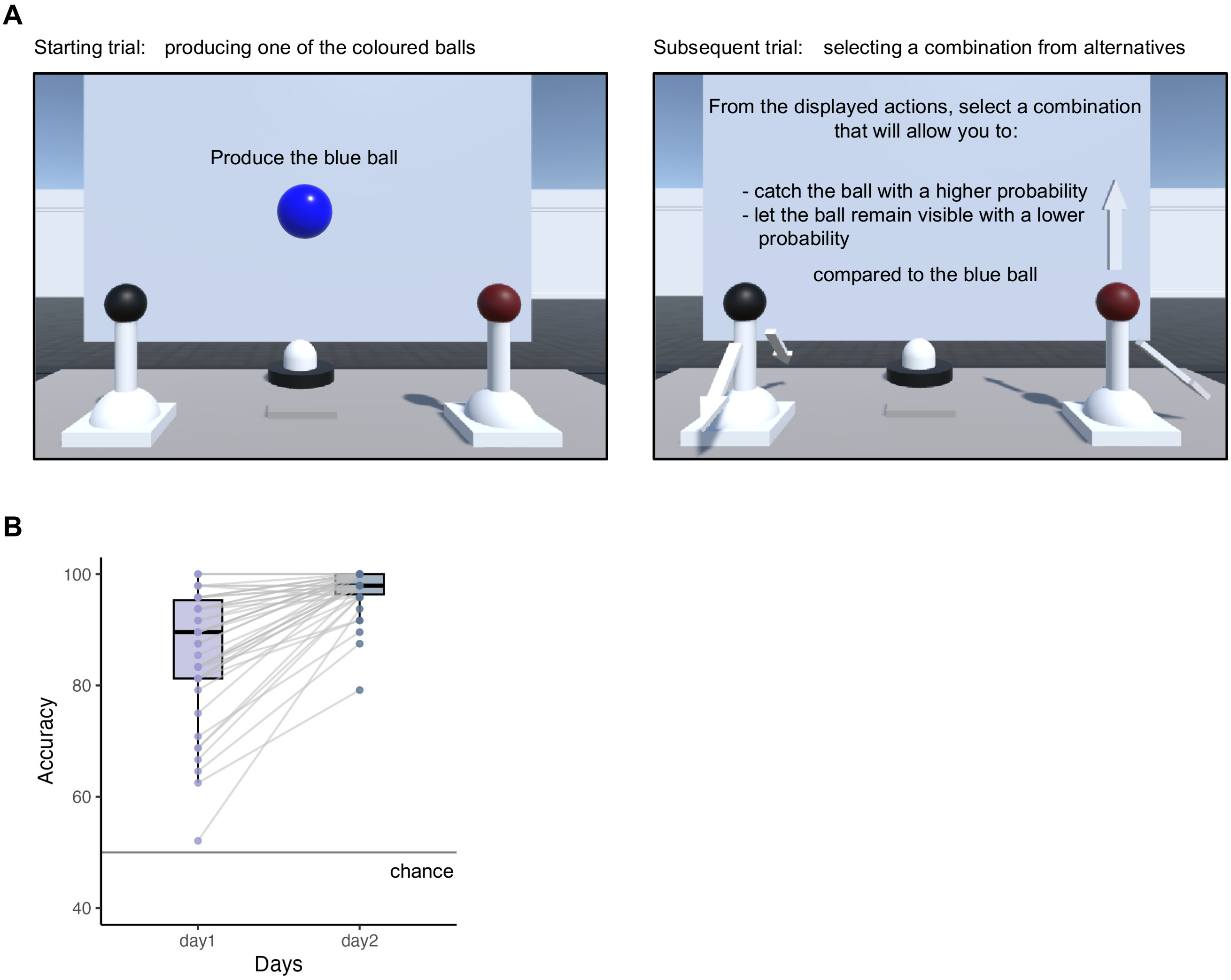

Supplementary Figure 1: Goal-directed Action Task. A In this VR multiple-choice task, participants completed pairs of trials. In the first, starting trial of each pair, they were instructed to produce one of the coloured balls, e.g. the blue ball. The second subsequent trial asked participants to use the previously produced coloured ball, in this case the blue ball, as a reference point and to select a correct combination of actions from the cued alternatives in order to achieve task-relevant outcomes. The example instructions are shown in the figures. **B** Behavioural performance over the two days of training, demonstrating that participants could correctly identify and perform a combination of actions from the cued alternatives to elicit the desired outcomes. Dots represent data from *n* = 46 participants. boxplots show median and upper/lower quartile with whiskers extending to the most extreme data point within 1.5 interquartile ranges above/below the quartiles.

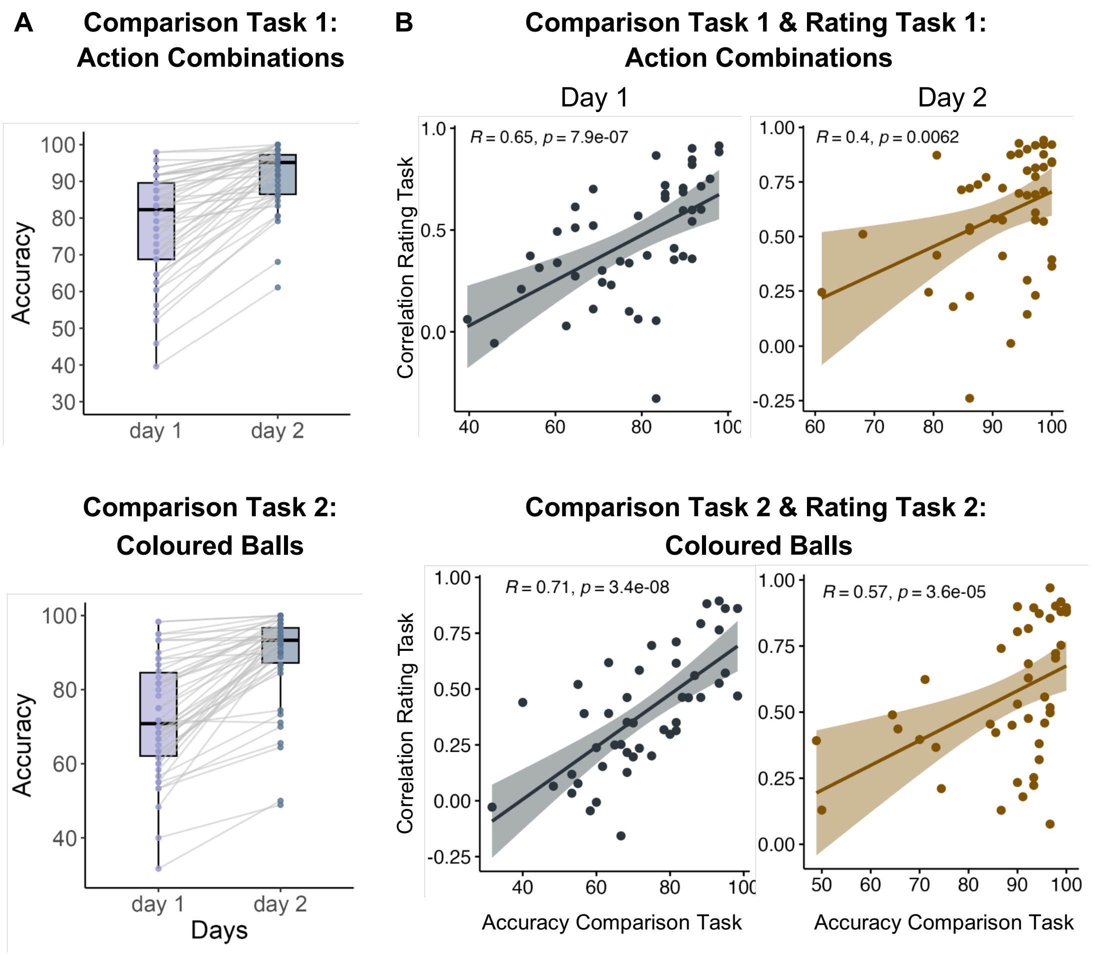

Supplementary Figure 2: Behavioural performance in the two Comparison Tasks and correlation between Comparison and Rating Tasks. A Overall performance over the two days of training in the Comparison tasks with action combinations (plot above) and coloured balls (plot below). Dots represent data from $n = 46$ participants. boxplots show median and upper/lower quartile with whiskers extending to the most extreme data point within 1.5 interquartile ranges above/below the quartiles. **B** We examined the Spearman's correlation between performance in the Comparison and Rating Tasks with action combinations (plots above) and coloured balls (plots below) over the two training days. Accuracy in the Comparison Tasks was correlated with the correlation values obtained by matching the similarity estimates from the Rating Tasks to the action-outcome space (see Figure 2A,C and Methods). Dots represent data from $n = 46$ participants. Line represents a trend line, with shaded regions as the 95% confidence interval.

Non-cardinal directions

Angle	30°	60°	120°	150°	210°	240°	300°	330°
2 x Dist	4.5	4.5	4.5	4.5	4.5	4.5	4.5	4.5
4 x Dist	3.6	3.6	3.6	3.6	3.6	3.6	3.6	3.6
6 x Dist	2.2	2.2	2.2	2.2	2.2	2.2	2.2	2.2
Mean (Count x Dist)	3.0	3.0	3.0	3.0	3.0	3.0	3.0	3.0

Cardinal directions

Angle	0°	90°	180°	270°
3 x Dist	4.0	4.0	4.0	4.0
4 x Dist	3.0	3.0	3.0	3.0
5 x Dist	2.0	2.0	2.0	2.0
Mean (Count x Dist)	2.8	2.8	2.8	2.8

Supplementary Figure 3. Distribution of distances covered by each direction in the abstract action-outcome space. The top panel shows the distances covered by the non-cardinal directions, while the bottom panel shows the distances for the cardinal directions. The first column of both panels shows the number of times each of the three distances was covered by each direction. It also shows the mean distance calculated for each direction based on the frequency of every distance travelled by that direction. For example, the average of 2 x 4.5, 4 x 3.6, 6 x 2.2 is 3. Other columns show that the distances in both cardinal and non-cardinal directions, as well as their means, were closely matched.

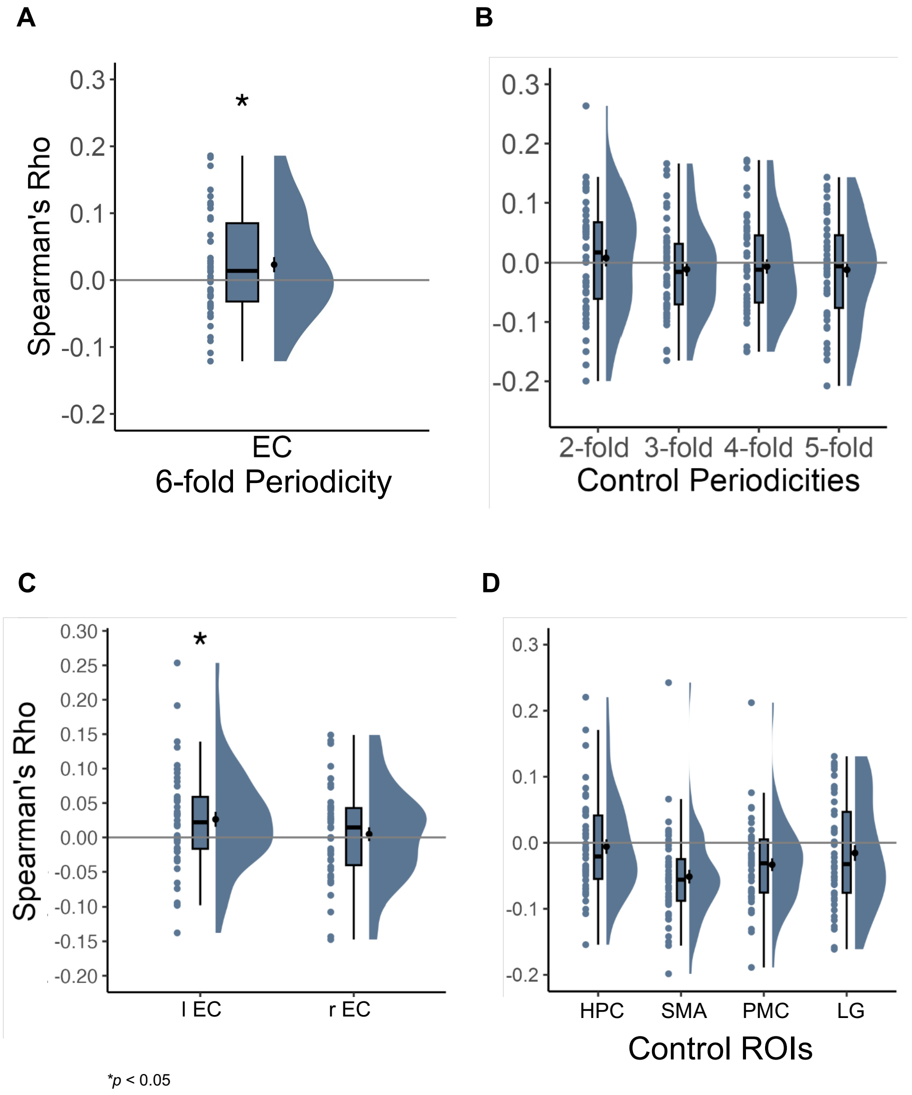

Supplementary Figure 4. Cross-validated representational similarity analysis (RSA) for the grid-like representation of the abstract action-outcome space. A Entorhinal cortex (EC) exhibited a significant 6-fold periodicity of pattern similarity (t(45)= 2.04, p=0.023, Cohen's d= 0.30, 95% CI= [0.0002, 0.045]; one-tailed t-test). **B** There was no statistically significant effect in control periodicities (2-fold: t(45)= 0.56, p= 0.291, 95% CI= [-0.02, 0.036]; 3-fold: t(45)= -0.96, p=0.83, 95% CI= [-0.034, 0.012]; 4-fold: t(45)= -0.53, p= 0.696, 95% CI= [-0.03, 0.017]; 5-fold: t(45)= -0.93, p= 0.826, 95% CI= [-0.037, 0.013]; one-tailed t-test). Only four of these are shown in the figure, as the 7-fold and 8-fold periodicities are equivalent to the 5-fold and 4-fold periodicities due to the size of the action-outcome space. In addition, we found no statistically significant relation between the similarity of the directions within the 2D abstract action-outcome space and their starting positions (t(45)= 0.82; p= 0.205, 95% CI= [-0.015, 0.036]; one-tailed t-test), ending positions (t(45)= -0.08; p= 0.525, 95% CI= [-0.029, 0.027]; one-tailed t-test), or a model combining both starting and ending positions in that space (t(45)= 0.49, p= 0.314, 95% CI= [-0.026, 0.044]; one-tailed t-test; see Methods for details). **C** Pattern similarities were extracted separately for each hemisphere using ROI masks and showed a significant 6-fold periodicity effect in the left EC (t(45)= 2.41, p= 0.017, Cohen's d= 0.35, 95% CI= [0.004, 0.048]; one-tailed t-test; Bonferroni corrected for tests in both ROIs) but not in the right EC (t(45)= 0.47, p= 0.322, 95% CI= [-0.015, 0.024]; one-tailed t-test). **D** None of the

control ROIs showed the statistically significant 6-fold periodicity effect (HPC: $t(45) = -0.53$, $p = 0.705$, 95% CI= [-0.027, 0.016]; SMA: $t(45) = -4.97$, $p = 1$, 95% CI= [-0.072, -0.03]; PMC: $t(45) = -3.44$, $p = 0.999$, 95% CI= [-0.052, -0.013]; LG: $t(45) = -1.29$, $p = 0.897$, 95% CI= [-0.039, 0.008]; one-tailed t-tests). Dots represent data from $n = 46$ participants. boxplots show median and upper/lower quartile with whiskers extending to the most extreme data point within 1.5 interquartile ranges above/below the quartiles; black circles with error bars correspond to mean ± SEM; distributions depict probability density functions of data points. *$p < 0.05$.

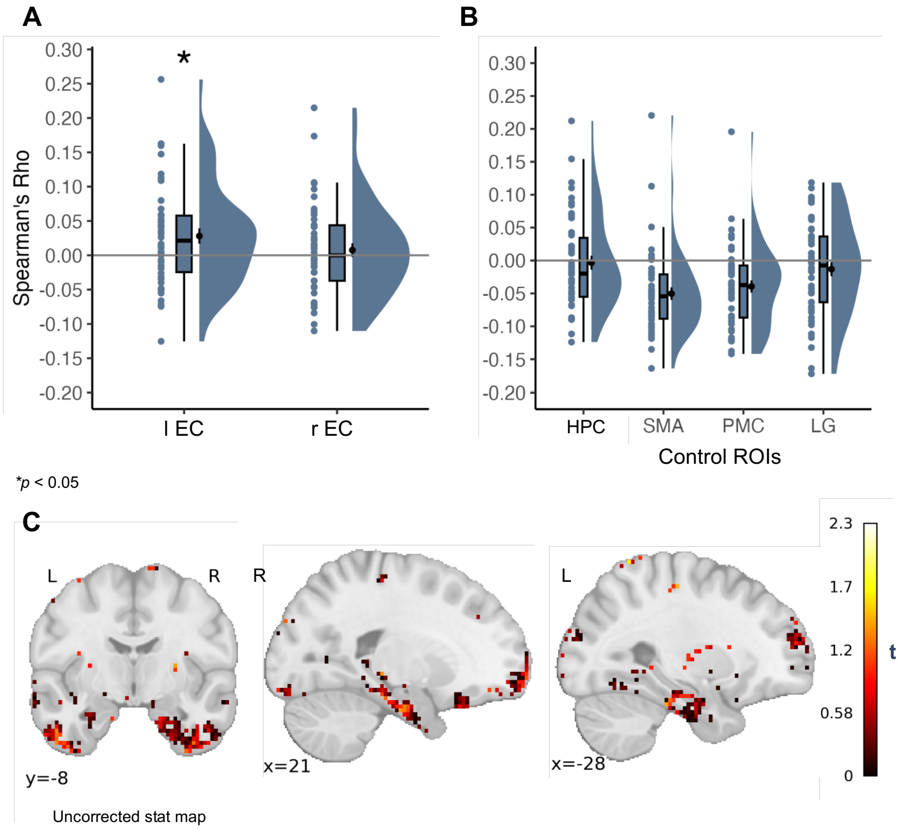
A
B
C
Spearman's Rho
0.30
0.25
0.20
0.15
0.10
0.05
0.00
-0.05
-0.10
-0.15
-0.20
*
l EC
r EC
HPC
SMA
PMC
LG
Control ROIs
*p < 0.05
L
R
R
L
y=-8
x=21
x=-28
2.3
1.7
1.2
0.58
0
t
Uncorrected stat map

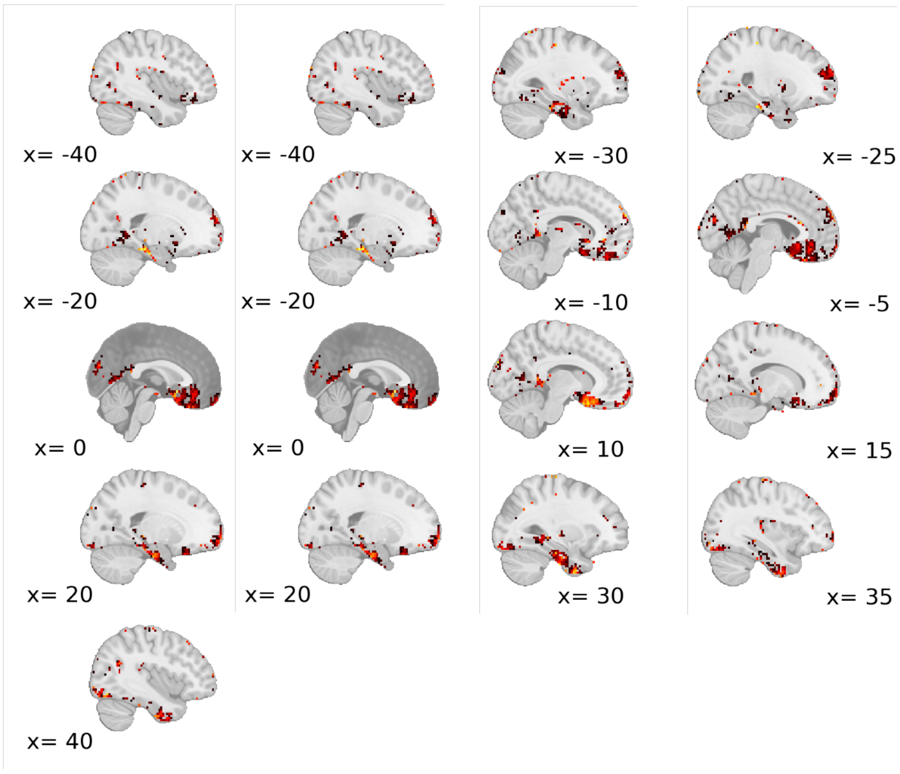
x= -40
x= -40
x= -30
x= -25
x= -20
x= -20
x= -10
x= -5
x= 0
x= 0
x= 10
x= 15
x= 20
x= 20
x= 30
x= 35
x= 40

Supplementary Figure 5: Grid-like representation of the abstract action-outcome space. A Representation similarity analysis (RSA) performed on the pattern similarities from the first Comparison Task (see Methods). Here, pattern similarities were extracted separately for each hemisphere using ROI masks and showed a significant 6-fold periodicity effect in the left EC (see Results). **B** No statistically significant 6-fold periodicity effect was observed in any of the control ROIs (HPC: t(45)= -0.31, p= 0.625, 95% CI= [-0.024, 0.017]; SMA: t(45)= -5.26, p= 1, 95% CI= [-0.069, -0.031]; PMC: t(45)= -4.25, p= 0.999, 95% CI= [-0.058, -0.02]; LG: t(45)= -1.22, p= 0.886, 95% CI= [-0.034, 0.008]**;** one-tailed t-test). **C** Whole-brain searchlight RSA using 5 voxel radius spheres revealed a cluster in bilateral EC (Uncorrected; MNI peak voxel coordinates: 27, 1.8, -48; peak voxel Spearman's rho = 1.78) as well as in the medial prefrontal cortex (Uncorrected; MNI peak voxel coordinates: 8, 29, -23; peak voxel Spearman's rho = 1.77). Whole-brain searchlight results are uncorrected for multiple comparisons. **A,B** Dots represent data from $n = 46$ participants. boxplots show median and upper/lower quartile with whiskers extending to the most extreme data point within 1.5 interquartile ranges above/below the quartiles; black circles with error bars correspond to mean ± SEM; distributions depict probability density functions of data points. $^*p < 0.05$; Bonferroni corrected for tests in both ROIs.

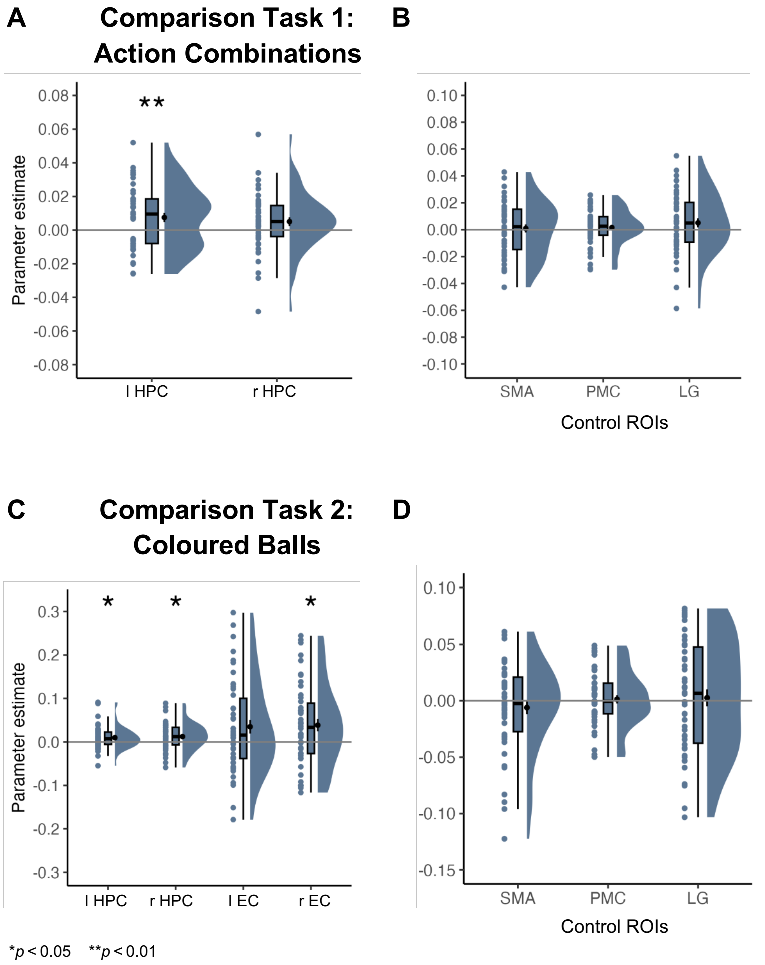

Supplementary Figure 6: Distance representations of the abstract action-outcome space. A The hippocampal BOLD response showed adaptation for trials with shorter distances between action combinations in the left hippocampus for the first Comparison Task (similar to the grid-like representation, see Supplementary Figure 4A; see Results). **B** None of the control ROIs showed the statistically significant adaptation effect of the BOLD signal to the distance between action combinations in the abstract action-outcome space (SMA: t(45)= 0.3, p= 0.37, 95% CI= [-0.005, 0.006]; PMC: t(45)= 0.8, p= 0.207, 95% CI= [-0.002, 0.005]; LG: t(45)= 1.52, p= 0.068, 95% CI= [-0.001, 0.011]; one-tailed t-test). **C** The adaptation effect in the second Comparison Task, for coloured balls positioned closer to each other in the action-outcome space, was present in both hemispheres for the HPC and only in the right hemisphere for the EC (Left HPC: t(45)= 2.46, p= 0.028, Cohen's d= 0.36, 95% CI= [0.001, 0.017]; Right HPC: t(45)= 2.69, p= 0.018, Cohen's d= 0.39, 95% CI= [0.003, 0.021]; Left EC: t(45)= 2.21, p= 0.061, 95% CI= [0.003, 0.066]; Right EC: t(45)= 2.73, p= 0.018, Cohen's d= 0.40, 95% CI= [0.01, 0.066]**;** one-tailed t-test). **D** The control ROIs including SMA, PMC and LG did not show the statistically significant adaptation effect (SMA: t(45)= -1.02, p= 0.84, 95% CI= [-0.018, 0.005]; PMC: t(45)= 0.34, p= 0.36, 95% CI= [-0.006, 0.008]; LG: t(45)= 0.35, p= 0.355, 95% CI= [-0.012, 0.017]; one-tailed t-test). Dots represent data from $n = 46$ participants. boxplots show median and upper/lower quartile with whiskers extending to the most extreme data point within 1.5 interquartile ranges above/below the quartiles; black circles with error bars correspond to mean ± SEM; distributions depict probability density functions of data points. *$p < 0.05$; **$p < 0.01$; The results for the HPC and EC ROIs are Bonferroni corrected for tests in multiple ROIs.

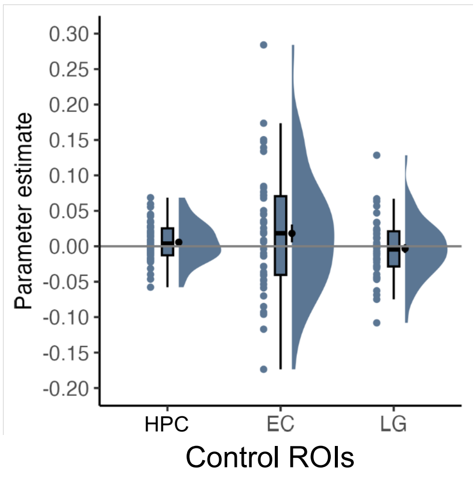

Supplementary Figure 7: Control analyses for the representation of individual actions. The shared actions between action combinations did not elicit the statistically significant modulation of BOLD response in the control ROIs (HPC: t(45)= 1.4**,** p= 0.162, 95% CI= [-0.002, 0.013]; EC: t(45)= 1.45, p= 0.148, 95% CI= [-0.007, 0.043]; LG: t(45)= -0.53, p= 0.606, 95% CI= [-0.015, 0.009]; two-tailed t-test). Dots represent data from $n = 46$ participants. boxplots show median and upper/lower quartile with whiskers extending to the most extreme data point within 1.5 interquartile ranges above/below the quartiles; black circles with error bars correspond to mean ± SEM; distributions depict probability density functions of data points.

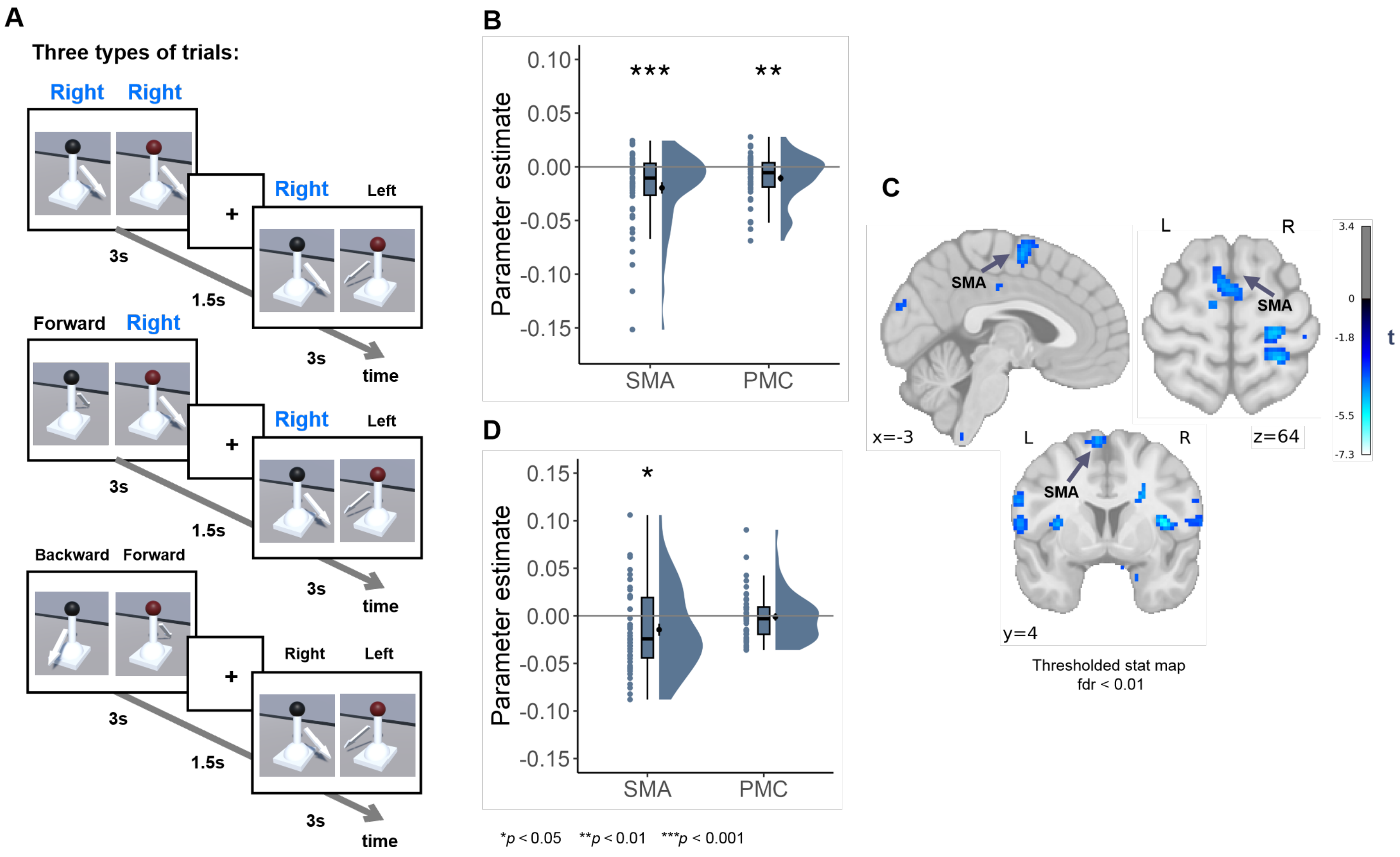

Supplementary Figure 8. Representation of individual actions in the SMA. A Trials from the first Comparison Task were categorized into three different trial types. The first type, shown in the top panel, consisted of trials where the two action combinations of a pair shared the common action, which occurred three times: in the current example of overlapping action plans, the common action would be moving the joystick to the right with both joysticks in the first action combination and with one joystick in the second action combination. The middle panel shows a second trial type, where the shared common action occurs only once in each of the combinations, shared by the same or by the different joysticks. The third trial type with non-overlapping action plans is shown on the bottom panel. **B** The logic of the analysis is the same as of the main analysis (see Methods). The constructed parametric regressor based on the three trial types (**A**) yielded significant results for both ROIs of interest (SMA: t(45)=-3.66, p<0.001, Cohen's d= -0.54, 95% CI= [-0.03, -0.008]; PMC: t(45)=-3.21, p=0.003, Cohen's d= -0.47, 95% CI= [-0.017, -0.003]; two-tailed t-test). **C** The action similarity-dependent increase in activity in the SMA was confirmed on the whole-brain level (FDR-corrected using a voxel-level threshold of p<0.01; MNI peak voxel coordinates: -2, -3, 61; peak voxel t(45) = -4.586; two-tailed test). **D** To fully disentangle actions from their outcomes, we performed a control analysis that differed from the previous analysis only in the types of trials entered as 'overlapping action plans'. Specifically, the analysis was restricted to the trials where the common action between the two action combinations was shared by different joysticks (e.g., joystick 1 in the first and joystick 2 in the second combination cued the movement to the right). The modulation of the BOLD activity remained statistically significant in the SMA (t(45)=-2.29, p=0.024, Cohen's d= -0.33, 95% CI= [-0.027, -0.001]; two-tailed t-test) but not in the PMC (t(45)=-0.29, p=0.775, 95% CI= [-0.008, 0.006]; two-tailed t-test), suggesting that the SMA represents action-related information. **B,D** Dots represent data from *n* = 46 participants. boxplots show median and upper/lower quartile with whiskers

extending to the most extreme data point within 1.5 interquartile ranges above/below the quartiles; black circles with error bars correspond to mean ± SEM; distributions depict probability density functions of data points. *$p < 0.05$; **$p < 0.01$; ***$p < 0.001$.

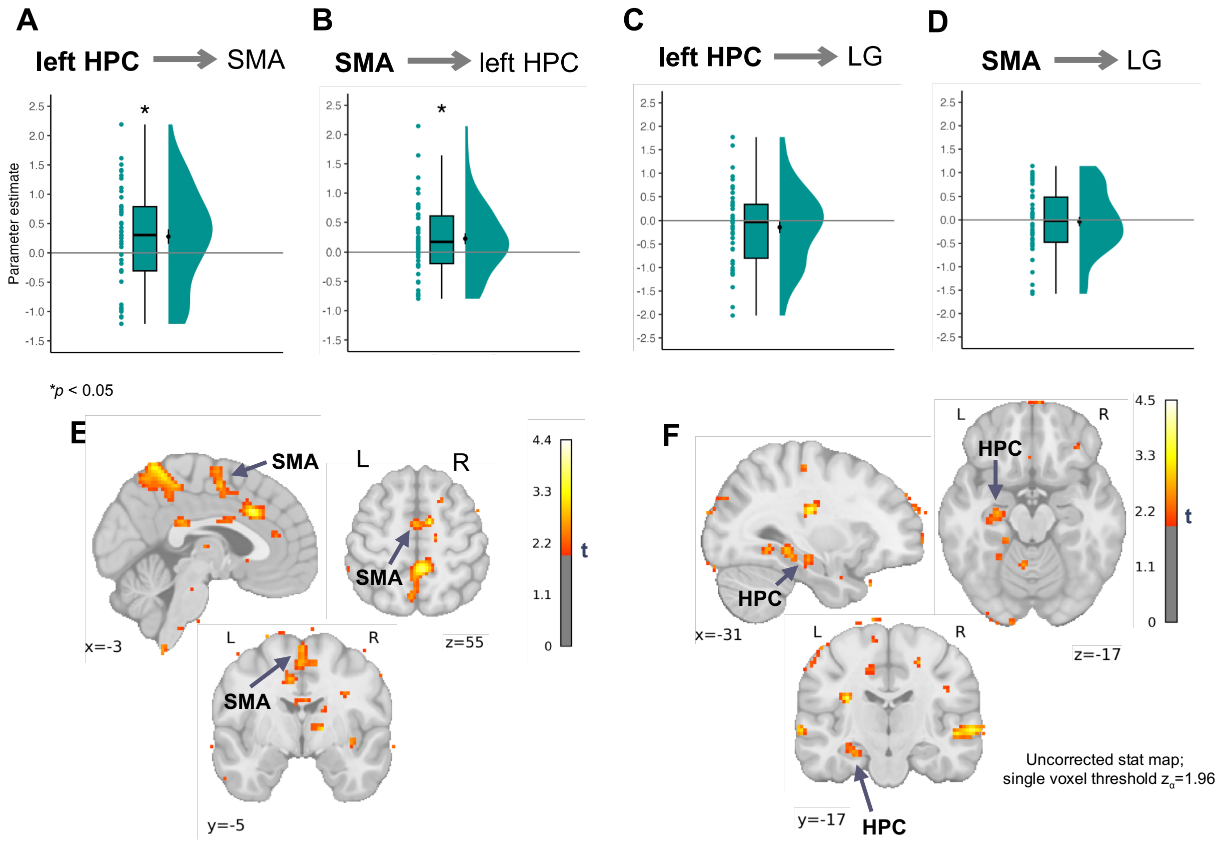

Supplementary Figure 9: Interaction between map-like representations in the hippocampus and individual action representations in SMA. Analyses were performed on the data from the first Comparison Task (see Methods). **A,B,C,D** left HPC used as a seed ROI to assess the connectivity with the SMA (HPC-SMA: t(45) = 2.27, p= 0.027, Cohen's d= 0.33, 95% CI= [0.032, 0.524]; two-tailed t-test) (**A**) and with the control ROI LG (HPC-LG: t(45)= -1.15, p= 0.252, 95% CI= [-0.387, 0.105]; two-tailed t-test) (**C**). SMA used as a seed ROI to assess the connectivity with the left HPC (SMA-HPC: t(45)= 2.4, p= 0.022; Cohen's d= 0.35, 95% CI= [0.035, 0.404]; two-tailed t-test) (**B**) and with the control ROI LG (SMA-LG: t(45)= -0.34, p=0.733, 95% CI= [-0.237, 0.167]; two-tailed t-test) (**D**). **E** Whole-brain connectivity map for the left HPC used as a seed. MNI peak voxel coordinates of SMA: 3, 1, 48; peak voxel t(45) = 3.175; two-tailed test. **F** Whole-brain connectivity map for the SMA used as a seed. MNI peak voxel coordinates of lHC: -26, -15, -18; peak voxel t(45) = 2.802; two-tailed test. Whole-brain data is uncorrected for multiple comparisons. Statistical significance threshold is defined at a single voxel level (z_α=1.96). **A,B,C,D** Dots represent data from $n = 46$ participants. boxplots show median and upper/lower quartile with whiskers extending to the most extreme data point within 1.5 interquartile ranges above/below the quartiles; black circles with error bars correspond to mean ± SEM; distributions depict probability density functions of data points. *$p < 0.05$.

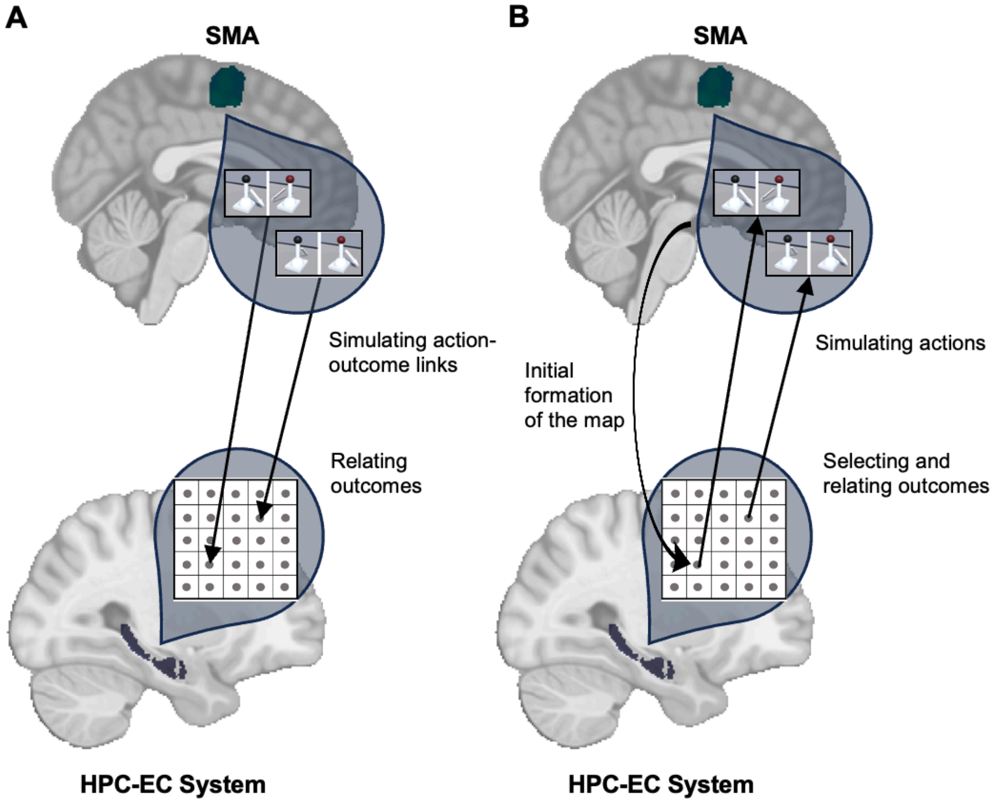

Supplementary Figure 10: Speculative schematic representation of alternative interaction models between SMA and HPC-EC System. The figure illustrates two possible speculative models of interaction between the SMA and the HPC-EC system. **A** The SMA might simulate individual action-outcome links using a forward model approach, allowing the HPC-EC system to position them within an abstract cognitive map for subsequent computation of relations between multiple action-outcome pairs. This model emphasizes a forward connection from SMA to HPC-EC system. **B** Alternatively, the hippocampal-entorhinal system might integrate information about individual action-outcome links from the SMA during the initial formation of the map (indicated by a curved arrow). Later, the HC-EC can act as an inverse model, by providing a map of pointers that, given a desired outcome, can retrieve the corresponding action representations from the SMA (indicated by straight arrows).

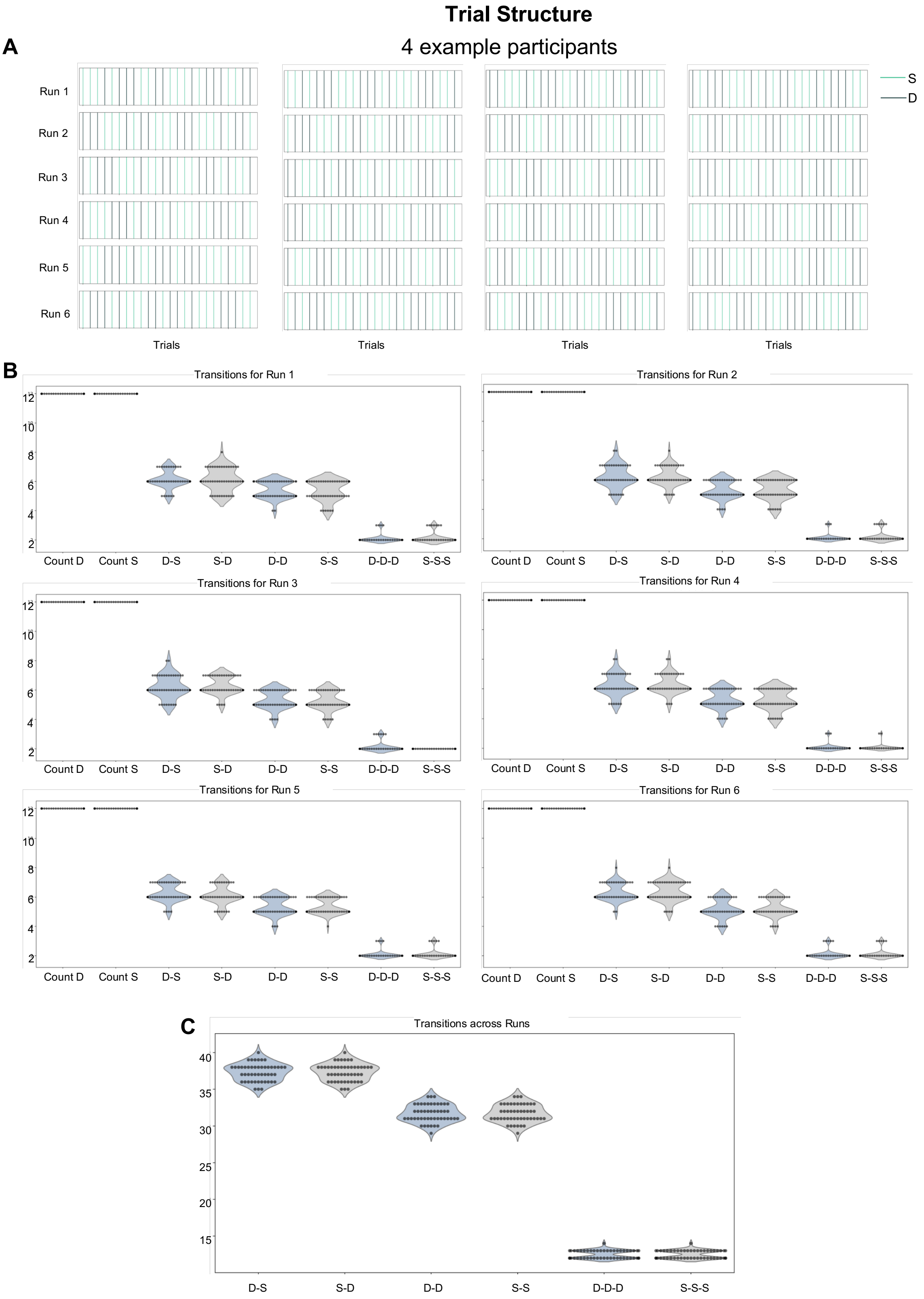

Supplementary figure 11. Trial structure of the first Comparison Task. A The trial structure was carefully designed to be balanced and optimized for the RSA analysis (see Methods, Randomization structure for the first Comparison Task). The distribution of trials with similar and dissimilar directions across runs (see Methods) for four example participants is shown for illustration purposes. Two

direction types (similar or dissimilar) were presented an equal number of times within each run (see panel **B**, Count D and Count S). In addition, the direction types were equally distributed within each half of the run, with the same number for both halves of the run. The vertical green lines represent trials with similar directions, whereas the dark grey lines represent trials with dissimilar directions. The trial structure of every run differed from all other runs by at least 10 elements. Furthermore, the trial structures of runs varied across participants **B,C** All possible transitions between the two direction types were considered and divided into categories of transitions, and the number of their occurrences was well balanced both within and across participants, with only minor deviations. For individual participants, the number of transitions from dissimilar to similar direction and vice versa (in other words, the number of times the dissimilar direction was followed by a similar direction and the similar direction was followed by a dissimilar direction, abbreviated as D-S and S-D) was almost equal in each run (**B**) and completely equal across all six runs (**C**). Following the same logic, the number of transitions from dissimilar to dissimilar direction (D-D) or from similar to similar direction (S-S) was almost equal in each run (**B**) and completely equal across all six runs (**C**). The same applies to the triple transitions (D-D-D and S-S-S). The black dots in the plot represent individual participants.

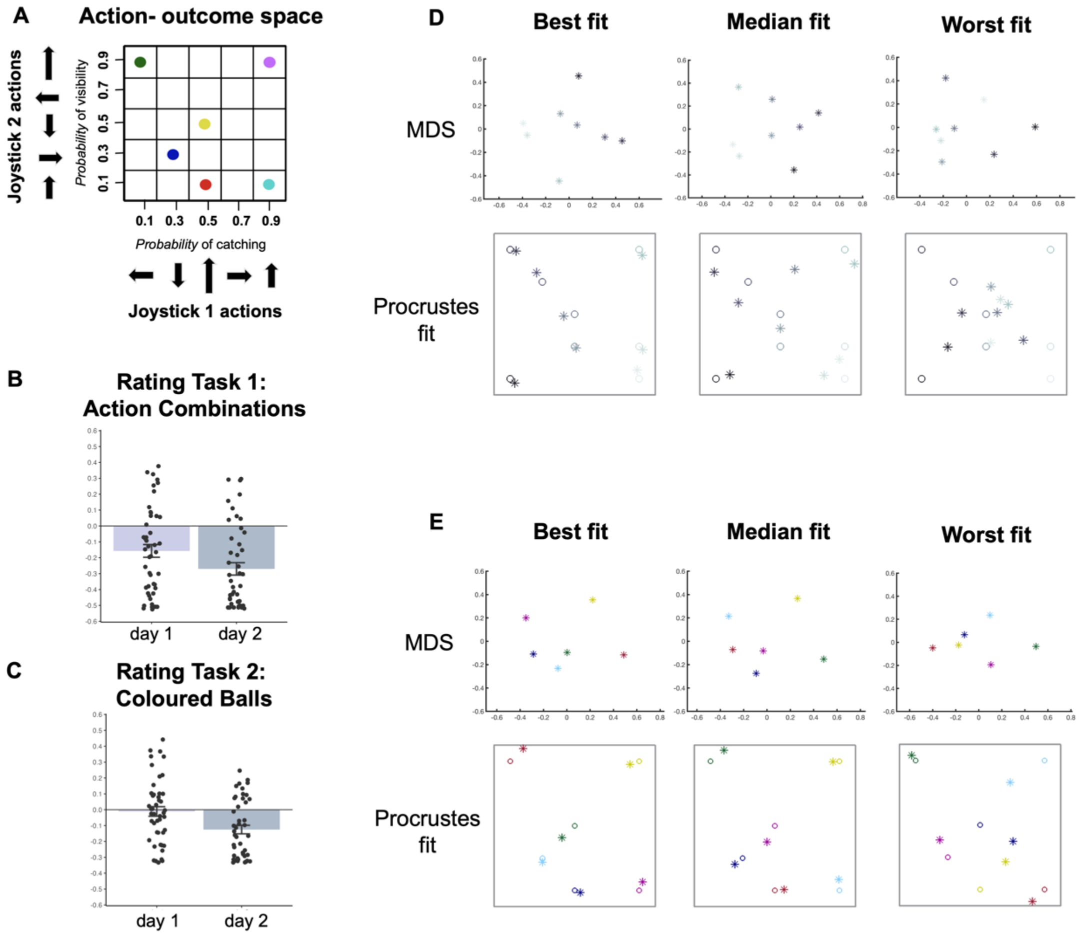

Supplementary Figure 12. Reconstructed map-like representation obtained from the two Rating Tasks. A Abstract action-outcome space. **B, C** The Procrustes distances obtained by fitting the multidimensional scaling (MDS) coordinates of the action combinations (**B**) and coloured balls (**C**) to the true coordinates of the respective stimuli in the action-outcome space were smaller than the critical distances obtained from the permutation tests (see Figure 2; see Methods). **D, E** The Procrustes distances from Rating Task 1 (**B**) and Rating Task 2 (**C**) were used to define three example participants with best, median and worst fit. The corresponding upper (**D**) and lower (**E**) panels demonstrate the data from day 2. **B,C** Dots represent data from $n = 46$ participants. The plots show mean values with error bars representing ± SEM.

Model RDMs

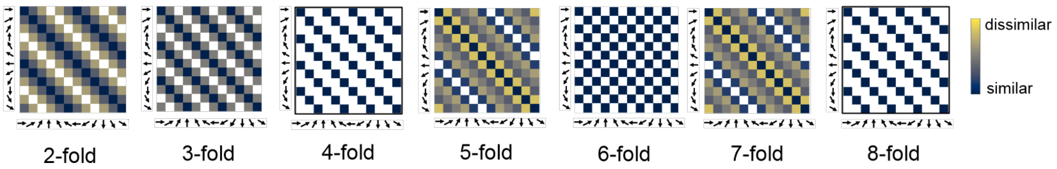

Periodicities

Supplementary Figure 13. Different models of pattern similarity values. To test for the representation of directions in the abstract action-outcome space, we created eight different models of pattern similarity between pairs of trials sampling different directions in the first Comparison Task. Each entry on the modeled representational dissimilarity matrices (RDMs) corresponds to a direction in the abstract space, indicated by a small black arrow, relating the first to the second action combination of a pair (see Figure 3A and Methods). We hypothesized that pattern similarity would follow a 6-fold periodicity in the entorhinal cortex, while the other models served as control periodicities for the analysis. The modeled periodicities correspond to a 180°, 120°, 90°, 72°, 60°, 51.43° and 45° modulation of pattern similarity values (see Figure 3 for results).

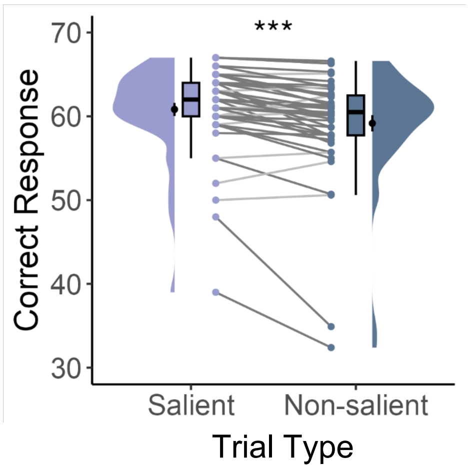

***p < 0.001

Supplementary Figure 14. Effects of landmark outcomes on the behavioural performance from the first Comparison Task. The behavioural data stems from the first scanning session, where participants performed 144 trials of comparisons between sequentially presented pairs of action combinations. Trials were divided in two categories according to whether any action combination in the pair was associated with landmark outcomes, i.e., coloured balls (salient), or whether neither combination had such an association (non-salient). For each participant, the number of trials with no association with coloured balls was subsampled to match the number of trials with an association (M=64.6, SD=2.78). The number of correct responses for both trial categories was recorded. The subsampling process was repeated 10 times, and correct responses were averaged across iterations. Accuracy was higher in trials linked to coloured balls ($t(45)= 4.17$, $p<0.001$, Cohen's d= 0.24, 95% CI= [0.127, 0.365]; two-tailed paired t-test), suggesting that the landmark outcomes of actions might facilitate the recall of other associated outcomes, namely the probability of catching the ball and its visibility. However, the means for salient and non-salient trial types are almost equal (M salient = 60.8, M non-salient = 59.2), and thus the interpretation of these results, albeit statistically significant in their difference, must be approached with caution. Dots represent data from $n = 46$ participants. boxplots show median and upper/lower quartile with whiskers extending to the most extreme data point within 1.5 interquartile ranges above/below the quartiles; black circles with error bars correspond to mean ± SEM; distributions depict probability density functions of data points. ***p < 0.001.

Supplementary Table 1: Significant clusters of the abstract distance-based BOLD adaptation analysis of the action combinations. Clusters surviving the whole-brain correction (two-sided t-test, FDR-corrected using a voxel-level threshold of p<0.01). Table displays MNI coordinates (X, Y, Z), statistical T values and atlas labels of peak voxels of the clusters. Atlas labels are based on the Juelich Histological Atlas (JHA) and Harvard-Oxford Cortical Structural Atlas (HOCSA). The labels were generated using FSL. Atlases are listed only if the labels were found for a given atlas. The letters after the cluster ID indicates its subcluster.

Cluster I	X	Y	Z	T value	Cluster Size (voxels)	Atlas label
1	-36.788	-35.468	12.25	6.668	1446	JHA:<br>26% GM Primary auditory cortex TE1.1 L, 18% WM A radiation L, 8% WM Optic radiation L, 6% GM Second somatosensory cortex / Parietal operculum OP1 L, 6% Inferior parietal lobule PFcm L, 2% GM Insula Ig1 L<br><br>HOCSA:<br>34% Planum Temporale, 2% Supramarginal Gyrus, po division, 1% Heschl's Gyrus (includes H1 and H2), 1% Parietal Operculum Cortex
1a	-44.252	-30.492	20.5	4.473		JHA:<br>61% GM Secondary somatosensory cortex / Parietal operculum OP1 L, 23% GM Inferior parietal lobule PF 18% GM Inferior parietal lobule PFcm L, 15% GM Prin auditory cortex TE1.1 L, 4% GM Primary auditory cort TE1.0 L, 2% GM Insula Ig2 L<br><br>HOCSA:<br>65% Parietal Operculum Cortex, 7% Central Opercula Cortex, 2% Superior Temporal Gyrus, posterior divisio Planum Temporale, 1% Heschl's Gyrus (includes H1 a
2	17.947	49.123	37.0	5.881	3881	HOCSA:<br>84% Frontal Pole
2a	25.411	51.611	23.25	4.820		HOCSA:<br>82% Frontal Pole
3	57.755	-28.004	23.25	5.842	8239	JHA:<br>49% GM Inferior parietal lobule PFcm R, 32% GM Se somatosensory cortex / Parietal operculum OP1 R, 12 Inferior parietal lobule PF R, 4% GM Inferior parietal lo PFt R, 1% GM Inferior parietal lobule PFop R<br><br>HOCSA:<br>33% Parietal Operculum Cortex, 21% Supramarginal anterior division, 13% Planum Temporale, 1% Supran Gyrus, posterior division
3a	67.707	-40.444	26.0	5.351		JHA:<br>22% GM Inferior parietal lobule PF R, 2% GM Inferior lobule Pga R<br><br>HOCSA:<br>34% Supramarginal Gyrus, posterior division, 2% Ang Gyrus, 1% Superior Temporal Gyrus, posterior divisio
3b	67.707	-47.908	9.5	5.284		JHA:<br>3% GM Inferior parietal lobule Pga R<br><br>HOCSA:<br>24% Middle Temporal Gyrus, temporooccipital part, 1 Angular Gyrus, 4% Supramarginal Gyrus, posterior di
3c	65.219	-32.98	28.75	5.212		JHA:<br>83% GM Inferior parietal lobule PF R, 17% GM Inferio parietal lobule PFcm R<br><br>HOCSA:<br>33% Supramarginal Gyrus, anterior division, 13% Sup Temporal Gyrus, posterior division, 11% Supramargin Gyrus, posterior division, 9% Planum Temporale, 7% Operculum Cortex
4	-24.348	49.123	37.0	5.640	731	HOCSA:

						77% Frontal Pole
5	52.779	-0.636	9.5	5.595	2059	JHA:<br>25% GM Secondary somatosensory cortex / Parietal operculum OP4 R, 14% GM Secondary somatosenso cortex / Parietal operculum OP3 R, 2% GM Inferior pa lobule PFop R<br><br>HOCSA:<br>34% Central Opercular Cortex, 9% Precentral Gyrus, Inferior Frontal Gyrus, pars opercularis
5a	45.315	6.827	6.75	4.34		JHA:<br>6% GM Secondary somatosensory cortex / Parietal operculum OP4 R, 1% GM Broca's area BA44 R<br><br>HOCSA:<br>54% Central Opercular Cortex, 5% Frontal Operculum 2% Precentral Gyrus, 1% Inferior Frontal Gyrus, pars opercularis
6	50.291	-18.052	-12.5	5.587	1038	JHA:<br>1% GM Insula Id1 R<br><br>HOCSA:<br>51% Middle Temporal Gyrus, posterior division, 20% Temporal Gyrus, posterior division, 2% Middle Tempo Gyrus, anterior division
6a	57.755	-13.076	-7.0	4.3		HOCSA:<br>43% Superior Temporal Gyrus, posterior division, 28% Temporal Gyrus, posterior division, 7% Middle Tempo Gyrus, anterior division, 3% Superior Temporal Gyrus, anterior division
6b	65.219	-18.052	-7.0	3.882		HOCSA:<br>59% Middle Temporal Gyrus, posterior division, 19% Temporal Gyrus, posterior division, 2% Middle Tempo Gyrus, anterior division
7	-39.276	1.851	15.0	5.542	800	JHA:<br>6% GM Broca's area BA44 L<br><br>HOCSA:<br>30% Central Opercular Cortex
8	-1.956	-18.052	42.5	5.147	2893	JHA:<br>9% GM Primary motor cortex BA4a L, 6% GM Premot cortex BA6 L<br><br>HOCSA:<br>65% Cingulate Gyrus, posterior division, 20% Cingula Gyrus, anterior division, 7% Precentral Gyrus
8a	0.531	-3.124	45.25	4.785		JHA:<br>36% GM Premotor cortex BA6 L<br><br>HOCSA:<br>43% Cingulate Gyrus, anterior division, 35% Juxtapos Lobule Cortex (formerly Supplementary Motor Cortex) Cingulate Gyrus, posterior division
8b	7.995	-20.54	42.5	4.21		JHA: 26% GM Premotor cortex BA6 R<br><br>HOCSA: 61% Cingulate Gyrus, posterior division, 9% Precentral Gyrus, 7% Cingulate Gyrus, anterior divisio
8c	-11.908	-10.588	50.75	4.017		JHA: 43% GM Premotor cortex BA6 L<br><br>HOCSA: 17% Juxtapositional Lobule Cortex (formerly Supplementary Motor Cortex), 1% Precentral Gyrus
9	-9.42	-90.204	34.25	5.022	527	JHA: 9% GM Superior parietal lobule 7P L, 2% GM Vi cortex V1 BA17 L, 1% GM Visual cortex V2 BA18 L, 1 Superior parietal lobule 7A L<br><br>HOCSA: 44% Occipital Pole, 19% Lateral Occipital Co superior division, 5% Cuneal Cortex
10	50.291	39.171	-1.5	4.894	1055	JHA: 11% GM Broca's area BA45 R

						HOCSA: 73% Frontal Pole, 10% Inferior Frontal Gyrus triangularis
10a	57.755	36.683	1.25	4.555		JHA: 39% GM Broca's area BA45 R<br><br>HOCSA: 14% Frontal Pole, 6% Inferior Frontal Gyrus, triangularis
11	30.387	-23.028	-20.75	4.87	425	JHA:<br>37% GM Hippocampus subiculum R, 21% GM Hippoc cornu ammonis R, 7% WM Cingulum R, 2% WM Optic radiation R, 2% GM Hippocampus dentate gyrus R<br><br>HOCSA: 32% Parahippocampal Gyrus, posterior divis Parahippocampal Gyrus, anterior division, 1% Tempo Fusiform Cortex, posterior division
12	-11.908	-37.956	42.5	4.84	459	JHA:<br>17% GM Superior parietal lobule 5Ci L, 5% GM Super parietal lobule 5M L<br><br>HOCSA:<br>29% Precuneous Cortex, 29% Cingulate Gyrus, poste division, 6% Precentral Gyrus, 5% Postcentral Gyrus
13	-64.156	-23.028	17.75	4.819	1498	JHA:<br>49% GM Secondary somatosensory cortex / Parietal operculum OP1 L, 38% GM Inferior parietal lobule PF 1% GM Inferior parietal lobule PFt L<br><br>HOCSA:<br>25% Supramarginal Gyrus, anterior division, 24% Pos Gyrus, 12% Parietal Operculum Cortex, 9% Planum Temporale, 8% Central Opercular Cortex, 3% Superio Temporal Gyrus, posterior division
14	50.291	6.827	-40.0	4.733	1293	HOCSA:<br>57% Temporal Pole, 17% Inferior Temporal Gyrus, an division, 7% Middle Temporal Gyrus, anterior division
15	60.243	-13.076	15.0	4.688	442	JHA:<br>57% GM Secondary somatosensory cortex / Parietal operculum OP1 R, 42% GM Secondary somatosenso cortex / Parietal operculum OP4 R, 22% GM Inferior p lobule PFop R, 12% GM Secondary somatosensory c Parietal operculum OP3 R<br><br>HOCSA:<br>46% Central Opercular Cortex, 16% Postcentral Gyru Parietal Operculum Cortex, 2% Planum Temporale, 2 Planum Polare, 1% Heschl's Gyrus (includes H1 and Supramarginal Gyrus, anterior division, 1% Precentral
16	-31.812	-15.564	6.75	4.603	459	JHA:<br>4% GM Insula Ig2 L, 2% GM Secondary somatosenso cortex / Parietal operculum OP3 L<br><br>HOCSA:<br>3% Insular Cortex
17	10.483	-85.228	37.0	4.505	408	JHA:<br>1% GM Visual cortex V2 BA18 R<br><br>HOCSA:<br>29% Cuneal Cortex, 25% Occipital Pole, 9% Lateral O Cortex, superior division, 1% Precuneous Cortex
18	30.387	-40.444	67.25	4.457	323	JHA:<br>56% GM Primary somatosensory cortex BA1 R, 34% Primary somatosensory cortex BA3b R, 21% GM Prim somatosensory cortex BA2 R, 10% GM Superior parie lobule 7PC R, 10% GM Primary motor cortex BA4p R GM Premotor cortex BA6 R, 4% GM Primary motor co BA4a R, 3% WM Corticospinal tract R, 1% GM Superi parietal lobule 5M R, 1% GM Superior parietal lobule

						HOCSA:<br>38% Superior Parietal Lobule, 27% Postcentral Gyrus
18a	27.899	-37.956	59.0	3.851		JHA:<br>52% GM Primary somatosensory cortex BA2 R, 45% [illegible]<br>Primary somatosensory cortex BA3b R, 10% GM Prim<br>somatosensory cortex BA1 R, 10% GM Primary motor<br>BA4p R, 7% GM Superior parietal lobule 7A R, 5% G[illegible]<br>Superior parietal lobule 7PC R, 3% GM Superior parie<br>lobule 5L R, 1% GM Superior parietal lobule 5M R<br><br>HOCSA:<br>45% Postcentral Gyrus, 22% Superior Parietal Lobule
19	37.851	29.219	-18.0	4.445	170	HOCSA:<br>69% Frontal Orbital Cortex, 23% Frontal Pole
20	22.923	-55.372	-12.5	4.366	425	JHA:<br>4% GM Visual cortex V3V R, 1% GM Visual cortex V4<br>GM Visual cortex V2 BA18 R<br><br>HOCSA:<br>48% Lingual Gyrus, 36% Temporal Occipital Fusiform<br>4% Occipital Fusiform Gyrus
21	-1.956	-13.076	70.0	4.105	323	JHA:<br>76% GM Premotor cortex BA6 L, 16% GM Primary mo<br>cortex BA4a L<br><br>HOCSA:<br>32% Precentral Gyrus, 26% Juxtapositional Lobule<br>Cortex(formerly Supplementary Motor Cortex)
21a	-4.444	-10.588	61.75	3.912		JHA:<br>94% GM Premotor cortex BA6 L, 4% GM Primary mot<br>cortex BA4a L, 2% WM Corticospinal tract L<br><br>HOCSA:<br>63% Juxtapositional Lobule Cortex (formerly Supplem<br>Motor Cortex), 10% Precentral Gyrus
22	-46.74	-10.588	20.5	4.046	170	JHA:<br>38% GM Secondary somatosensory cortex / Parietal<br>operculum OP3 L, 31% GM Secondary somatosensor<br>/ Parietal operculum OP4 L, 8% GM Secondary<br>somatosensory cortex / Parietal operculum OP1 L, 3%<br>Primary somatosensory cortex BA3a L<br><br>HOCSA: 13% Central Opercular Cortex

Supplementary Table 2: Significant clusters of the abstract distance-based BOLD adaptation analysis of the landmark outcomes of actions. Clusters surviving the whole-brain correction (two-sided t-test, FDR-corrected using a voxel-level threshold of p<0.01). Table displays MNI coordinates (X, Y, Z), statistical T values and atlas labels of peak voxels of the clusters. Atlas labels are based on the Juelich Histological Atlas (JHA) and Harvard-Oxford Cortical Structural Atlas (HOCSA). The labels were generated using FSL. Atlases are listed only if the labels were found for a given atlas. The letters after the cluster ID indicates its subcluster.

Cluster	X	Y	Z	T Value	Cluster Size (	Atlas label
1	7.995	59.075	9.5	6.896	19610	HOCSA:<br>56% Frontal Pole, 3% Paracingulate Gyrus, 1% Superior Frontal G
1a	17.947	44.147	48.0	6.434		HOCSA:<br>66% Frontal Pole, 5% Superior Frontal Gyrus
1b	17.947	51.611	37.0	6.265		HOCSA:<br>83% Frontal Pole
1c	17.947	39.171	50.75	6.084		JHA:<br>6% GM Premotor cortex BA6 R<br><br>HOCSA:<br>53% Frontal Pole, 24% Superior Frontal Gyrus
2	32.875	6.827	9.5	5.983	16835	HOCSA:<br>28% Insular Cortex, 1% Central Opercular Cortex
2a	52.779	26.731	4.0	5.429		JHA:<br>50% GM Broca's area BA45 R, 1% GM Broca's area BA44 R<br><br>HOCSA:<br>38% Inferior Frontal Gyrus, pars triangularis, 8% Inferior Frontal Gy<br>pars opercularis, 5% Frontal Operculum Cortex, 2% Frontal Orbital
2b	45.315	19.267	-12.5	5.365		HOCSA:<br>36% Frontal Orbital Cortex, 9% Temporal Pole, 3% Insular Cortex,<br>Frontal Operculum Cortex
2c	35.363	-5.612	4.0	5.344		JHA:<br>8% GM Secondary somatosensory cortex / Parietal operculum OP3<br>GM Secondary somatosensory cortex / Parietal operculum OP2 R<br><br>HOCSA:<br>27% Insular Cortex
3	-41.764	9.315	-4.25	5.86	8170	HOCSA:<br>53% Insular Cortex, 10% Central Opercular Cortex, 5% Frontal Op<br>Cortex
3a	-36.788	-13.076	4.0	5.396		JHA:<br>12% GM Secondary somatosensory cortex / Parietal operculum OP<br>GM Insula Ig2 L, 3% GM Insula Id1 L<br><br>HOCSA:<br>52% Insular Cortex
3b	-39.276	-23.028	1.25	5.291		JHA:<br>45% WM Acoustic radiation L, 37% GM Insula Id1 L, 35% GM Insu<br>25% GM Primary auditory cortex TE1.1 L, 10% GM Primary auditor<br>TE1.0 L, 6% GM Insula Ig1 L<br><br>HOCSA:<br>36% Heschl's Gyrus (includes H1 and H2), 20% Planum Polare, 2%<br>Cortex
3c	-36.788	-3.124	-1.5	5.129		JHA:<br>1% WM Inferior occipito-frontal fascicle L<br><br>HOCSA:<br>24% Insular Cortex
4	57.755	-35.468	34.25	5.533	6587	JHA:

						63% GM Inferior parietal lobule PF R, 20% GM Inferior parietal lobu PFcm R, 16% GM Inferior parietal lobule PFm R, 12% GM Anterior parietal sulcus hIP2 R<br>HOCSA:<br>30% Supramarginal Gyrus, posterior division, 14% Parietal Opercu Cortex, 11% Supramarginal Gyrus, anterior division, 4% Planum Temporale, 2% Angular Gyrus
4a	60.243	-45.42	31.5	5.224		JHA:<br>78% GM Inferior parietal lobule PFm R, 20% GM Inferior parietal lo Pga R, 16% GM Inferior parietal lobule PF R<br>HOCSA:<br>45% Angular Gyrus, 43% Supramarginal Gyrus, posterior division
4b	52.779	-57.86	39.75	4.962		JHA:<br>73% GM Inferior parietal lobule Pga R, 30% GM Inferior parietal lob PGp R, 6% GM Inferior parietal lobule PFm R, 1% GM Anterior intra parietal sulcus hIP1 R<br>HOCSA:<br>41% Angular Gyrus, 36% Lateral Occipital Cortex, superior division
4c	60.243	-55.372	42.5	4.873		JHA:<br>4% GM Inferior parietal lobule Pga R, 3% GM Inferior parietal lobule R, 1% GM Inferior parietal lobule PGp R<br>HOCSA:<br>20% Angular Gyrus, 9% Lateral Occipital Cortex, superior division
5	-24.348	-5.612	-20.75	5.348	1702	JHA:<br>88% GM Amygdala_laterobasal group L, 28% GM Hippocampus co ammonis L, 25% GM Amygdala_superficial group L, 8% GM Amygdala_centromedial group L, 4% GM Hippocampus subiculum
6	7.995	-20.54	42.5	5.292	6758	JHA:<br>26% GM Premotor cortex BA6 R<br>HOCSA:<br>61% Cingulate Gyrus, posterior division, 9% Precentral Gyrus, 7% Cingulate Gyrus, anterior division
6a	5.507	-32.98	50.75	4.856		JHA:<br>48% GM Primary motor cortex BA4a R, 42% GM Superior parietal l 5M R, 24% GM Superior parietal lobule 5Ci R, 7% WM Corticospina R, 4% GM Premotor cortex BA6 R<br>HOCSA:<br>36% Precentral Gyrus, 22% Cingulate Gyrus, posterior division, 12 Postcentral Gyrus, 8% Precuneous Cortex
6b	-6.932	-35.468	50.75	4.557		JHA:<br>44% GM Superior parietal lobule 5M L, 21% GM Primary motor cor BA4a L, 10% GM Superior parietal lobule 5Ci L, 6% WM Corticospi L<br>HOCSA:<br>29% Precentral Gyrus, 21% Precuneous Cortex, 15% Postcentral G 12% Cingulate Gyrus, posterior division
6c	-14.396	-32.98	39.75	4.473		JHA:<br>46% GM Superior parietal lobule 5Ci L, 2% GM Superior parietal lo L<br>HOCSA:<br>29% Cingulate Gyrus, posterior division, 29% Precentral Gyrus, 11 Precuneous Cortex, 2% Postcentral Gyrus
7	27.899	-8.1	-18.0	5.155	1787	JHA:<br>79% GM Amygdala_laterobasal group R, 74% GM Hippocampus c ammonis R, 15% GM Hippocampus dentate gyrus R, 5% GM Amygdala_superficial group R, 3% GM Hippocampus subiculum R, Amygdala_centromedial group R
8	52.779	1.851	-34.5	4.946	987	HOCSA:<br>40% Middle Temporal Gyrus, anterior division, 14% Inferior Tempo Gyrus, anterior division, 6% Temporal Pole, 2% Middle Temporal G posterior division
9	-59.18	-28.0	15.0	4.882	1225	JHA:<br>36% GM Secondary somatosensory cortex / Parietal operculum OP 24% GM Inferior parietal lobule PFop L, 2% GM Inferior parietal lob PFcm L, 2% GM Inferior parietal lobule PF L<br>HOCSA:<br>46% Parietal Operculum Cortex, 30% Planum Temporale, 4% Cent Opercular Cortex, 4% Supramarginal Gyrus, anterior division, 1% S Temporal Gyrus, posterior division

10	-24.348	49.123	37.0	4.663	561	HOCSA:<br>77% Frontal Pole
11	-6.932	36.683	-4.25	4.337	1004	HOCSA:<br>56% Cingulate Gyrus, anterior division, 17% Paracingulate Gyrus, [illegible] Subcallosal Cortex, 1% Frontal Medial Cortex
11a	3.019	29.219	-7.0	4.221		HOCSA:<br>56% Subcallosal Cortex, 18% Cingulate Gyrus, anterior division, 6[illegible] Paracingulate Gyrus, 1% Frontal Medial Cortex
12	22.923	-42.932	56.25	4.302	442	JHA:<br>77% GM Primary somatosensory cortex BA2 R, 37% GM Superior [illegible] lobule 7PC R, 26% GM Superior parietal lobule 5L R, 15% GM Prim[illegible] somatosensory cortex BA1 R, 10% GM Primary motor cortex BA4p[illegible] GM Primary somatosensory cortex BA3b R, 6% GM Superior pariet[illegible] lobule 5M R, 1% GM Anterior intra-parietal sulcus hIP3 R<br>HOCSA:<br>22% Postcentral Gyrus, 20% Superior Parietal Lobule
12a	25.411	-40.444	64.5	3.749		JHA:<br>48% GM Primary somatosensory cortex BA2 R, 30% GM Primary somatosensory cortex BA3b R, 23% GM Primary somatosensory c[illegible] BA1 R, 10% GM Superior parietal lobule 7PC R, 8% GM Primary m[illegible] cortex BA4a R, 6% GM Superior parietal lobule 7A R, 6% GM Prim[illegible] motor cortex BA4p R, 4% GM Superior parietal lobule 5M R, 4% G[illegible] Superior parietal lobule 5L R, 2% GM Premotor cortex BA6 R, 1% [illegible] Corticospinal tract R<br>HOCSA:<br>35% Superior Parietal Lobule, 28% Postcentral Gyrus
13	40.339	-15.564	20.5	4.267	731	JHA:<br>61% GM Secondary somatosensory cortex / Parietal operculum O[illegible] 53% GM Secondary somatosensory cortex / Parietal operculum O[illegible] 13% GM Insula Ig2 R, 13% GM Secondary somatosensory cortex [illegible] operculum OP4 R, 10% GM Secondary somatosensory cortex / Pa[illegible] operculum OP1 R<br><br>HOCSA:<br>60% Central Opercular Cortex, 15% Parietal Operculum Cortex, 3[illegible] Cortex\n'
13a	50.291	-15.564	28.75	3.889		JHA:<br>36% GM Inferior parietal lobule PFop R, 15% GM Inferior parietal l[illegible] PFt R, 10% GM Secondary somatosensory cortex / Parietal opercu[illegible] OP4 R, 7% GM Secondary somatosensory cortex / Parietal opercu[illegible] OP3 R, 6% GM Secondary somatosensory cortex / Parietal opercu[illegible] OP1 R, 6% GM Primary somatosensory cortex BA3b R, 1% GM Pr[illegible] somatosensory cortex BA3a R<br>HOCSA:<br>10% Postcentral Gyrus, 3% Supramarginal Gyrus, anterior division
14	40.339	-35.468	15.0	4.225	204	JHA:<br>4% WM Optic radiation R<br>HOCSA:<br>25% Planum Temporale, 6% Supramarginal Gyrus, posterior divisi[illegible] Parietal Operculum Cortex
15	42.827	-42.932	-18.0	4.159	238	HOCSA:<br>34% Temporal Occipital Fusiform Cortex, 17% Inferior Temporal G[illegible] temporooccipital part, 10% Temporal Fusiform Cortex, posterior div[illegible] 1% Inferior Temporal Gyrus, posterior division
16	42.827	-10.588	53.5	4.094	255	JHA:<br>48% GM Premotor cortex BA6 R, 22% GM Primary motor cortex B[illegible] 16% WM Corticospinal tract R, 10% GM Primary somatosensory c[illegible] BA1 R, 6% GM Primary somatosensory cortex BA3b R<br>HOCSA:<br>48% Precentral Gyrus, 2% Postcentral Gyrus
17	65.219	-37.956	1.25	3.938	204	HOCSA:<br>36% Middle Temporal Gyrus, temporooccipital part, 30% Middle Te[illegible] Gyrus, posterior division, 12% Supramarginal Gyrus, posterior divis[illegible] Superior Temporal Gyrus, posterior division
18	35.363	31.707	48.0	3.846	221	HOCSA:<br>51% Middle Frontal Gyrus, 7% Frontal Pole, 2% Superior Frontal G[illegible]
19	-24.348	56.587	23.25	3.814	221	HOCSA: 81% Frontal Pole

Supplementary Table 3: Significant clusters of the action similarity-based BOLD adaptation analysis of the action combinations. Clusters surviving the whole-brain correction (two-sided t-test, FDR-corrected using a voxel-level threshold of p<0.01). Table displays MNI coordinates (X, Y, Z), statistical T values and atlas labels of peak voxels of the clusters. Atlas labels are based on the Juelich Histological Atlas (JHA) and Harvard-Oxford Cortical Structural Atlas (HOCSA). The labels were generated using FSL. Atlases are listed only if the labels were found for a given atlas. The letters after the cluster ID indicates its subcluster.

Cluster I	X	Y	Z	T Value	Cluster Size (vox	Atlas label
1	-56.692	-52.884	6.75	-7.433	16733	HOCSA:<br>54% Middle Temporal Gyrus, temporooccipital part, 9% Angu Gyrus, 7% Supramarginal Gyrus, posterior division, 3% Midd Temporal Gyrus, posterior division, 1% Lateral Occipital Cort inferior division
1a	-66.644	-30.492	4.0	-6.462		JHA:<br>2% GM Secondary somatosensory cortex / Parietal operculu L, 2% GM Inferior parietal lobule PF L<br><br>HOCSA:<br>69% Superior Temporal Gyrus, posterior division, 8% Middle Temporal Gyrus, posterior division, 1% Planum Temporale
1b	-61.668	-60.348	6.75	-6.381		JHA:<br>3% GM Inferior parietal lobule Pga L<br><br>HOCSA:<br>47% Middle Temporal Gyrus, temporooccipital part, 20% Lat Occipital Cortex, inferior division, 6% Angular Gyrus, 2% Late Occipital Cortex, superior division
1c	-59.18	-42.932	23.25	-6.26		JHA:<br>70% GM Inferior parietal lobule PF L, 35% GM Inferior pariet lobule PFm L, 14% GM Inferior parietal lobule PFcm L<br><br>HOCSA:<br>36% Supramarginal Gyrus, posterior division, 22% Parietal Operculum Cortex, 11% Planum Temporale, 4% Supramargi Gyrus, anterior division, 4% Superior Temporal Gyrus, poste division, 2% Angular Gyrus
2	-49.228	-75.276	-9.75	-6.38	5992	JHA:<br>4% GM Visual cortex V5 L<br><br>HOCSA:<br>77% Lateral Occipital Cortex, inferior division, 1% Occipital F Gyrus
2a	-41.764	-80.252	-9.75	-5.583		JHA:<br>7% GM Visual cortex V4 L<br><br>HOCSA:<br>67% Lateral Occipital Cortex, inferior division, 4% Occipital F Gyrus
2b	-44.252	-85.228	1.25	-4.945		JHA:<br>14% GM Visual cortex V4 L, HOCSA: 63% Lateral Occipital inferior division, 6% Occipital Pole, 2% Lateral Occipital Cort superior division
2c	-49.228	-60.348	-18.0	-4.128		HOCSA:<br>47% Inferior Temporal Gyrus, temporooccipital part, 18% Lat Occipital Cortex, inferior division, 14% Temporal Occipital Fu Cortex, 3% Occipital Fusiform Gyrus, 2% Middle Temporal G temporooccipital part
3	47.803	-80.252	6.75	-6.125	16971	JHA:<br>14% GM Visual cortex V5 R, 2% GM Inferior parietal lobule F 1% GM Visual cortex V4 R<br><br>HOCSA:<br>70% Lateral Occipital Cortex, inferior division, 5% Lateral Oc Cortex, superior division

3a	57.755	-30.492	37.0	-5.98		JHA:<br>52% GM Inferior parietal lobule PF R, 22% GM Inferior pariel lobule PFop R, 18% GM Inferior parietal lobule PFcm R, 10% Anterior intra-parietal sulcus hIP2 R, 8% GM Secondary somatosensory cortex / Parietal operculum OP1 R, 8% GM I parietal lobule PFt R, 5% GM Inferior parietal lobule PFm R<br><br>HOCSA:<br>51% Supramarginal Gyrus, anterior division, 9% Parietal Ope Cortex, 7% Supramarginal Gyrus, posterior division, 4% Pos Gyrus, 2% Planum Temporale
3b	62.731	-55.372	9.5	-5.676		JHA:<br>7% GM Inferior parietal lobule Pga R<br><br>HOCSA:<br>53% Middle Temporal Gyrus, temporooccipital part, 15% Ang Gyrus, 7% Lateral Occipital Cortex, inferior division, 2% Late Occipital Cortex, superior division
3c	55.267	-42.932	9.5	-5.547		JHA:<br>9% GM Inferior parietal lobule PFm R, 3% GM Inferior pariet Pga R<br><br>HOCSA:<br>36% Supramarginal Gyrus, posterior division, 22% Middle Te Gyrus, temporooccipital part, 10% Angular Gyrus, 4% Superi Temporal Gyrus, posterior division, 1% Middle Temporal Gyr posterior division
4	-59.18	14.291	12.25	-5.535	3251	JHA:<br>48% GM Broca's area BA44 L, 16% GM Broca's area BA45 GM Primary somatosensory cortex BA3b L<br><br>HOCSA:<br>27% Inferior Frontal Gyrus, pars opercularis, 7% Precentral
4a	-61.668	19.267	26.0	-4.76		JHA:<br>5% GM Broca's area BA45 L, 4% GM Broca's area BA44 L
4b	-51.716	16.779	20.5	-4.672		JHA:<br>44% GM Broca's area BA44 L, 13% GM Broca's area BA45<br><br>HOCSA:<br>58% Inferior Frontal Gyrus, pars opercularis, 5% Precentral 1% Inferior Frontal Gyrus, pars triangularis
4c	-49.228	9.315	9.5	-3.795		JHA:<br>27% GM Broca's area BA44 L, 2% GM Broca's area BA45 L<br><br>HOCSA:<br>45% Inferior Frontal Gyrus, pars opercularis, 11% Precentral 1% Frontal Operculum Cortex
5	-54.204	-0.636	42.5	-5.496	3557	JHA:<br>68% GM Premotor cortex BA6 L, 4% GM Broca's area BA44 WM Corticospinal tract L, 1% GM Primary somatosensory co BA1 L, 1% GM Primary motor cortex BA4a L<br><br>HOCSA:<br>75% Precentral Gyrus, 8% Middle Frontal Gyrus
5a	-41.764	1.851	50.75	-5.085		JHA:<br>24% GM Premotor cortex BA6 L<br><br>HOCSA:<br>36% Middle Frontal Gyrus, 26% Precentral Gyrus
5b	-41.764	-10.588	42.5	-4.011		JHA:<br>38% WM Corticospinal tract L, 38% GM Primary motor corte L, 33% GM Primary motor cortex BA4p L, 11% GM Primary somatosensory cortex BA3b L, 9% GM Premotor cortex BA6 GM Primary somatosensory cortex BA1 L<br><br>HOCSA:<br>30% Precentral Gyrus, 1% Postcentral Gyrus
6	42.827	-52.884	-15.25	-5.323	5055	HOCSA:<br>53% Temporal Occipital Fusiform Cortex, 10% Inferior Temp Gyrus, temporooccipital part

6a	32.875	-70.3	-15.25	-5.122		JHA:<br>48% GM Visual cortex V4 R, 3% GM Visual cortex V3V R<br><br>HOCSA:<br>75% Occipital Fusiform Gyrus, 2% Temporal Occipital Fusifo Cortex, 1% Lingual Gyrus, 1% Lateral Occipital Cortex, inferi division
6b	42.827	-65.324	-15.25	-4.949		JHA:<br>8% GM Visual cortex V4 R<br><br>HOCSA:<br>43% Occipital Fusiform Gyrus, 21% Lateral Occipital Cortex, division, 4% Temporal Occipital Fusiform Cortex, 3% Inferior Temporal Gyrus, temporooccipital part
6c	27.899	-50.396	-9.75	-4.693		JHA:<br>1% WM Optic radiation R<br><br>HOCSA: 54% Temporal Occipital Fusiform Cortex, 20% Ling Gyrus
7	-34.3	56.587	20.5	-5.096	476	HOCSA:<br>72% Frontal Pole
8	-36.788	41.659	37.0	-4.797	1174	HOCSA:<br>51% Frontal Pole, 16% Middle Frontal Gyrus
9	-6.932	4.339	67.25	-4.673	1957	JHA:<br>73% GM Premotor cortex BA6 L<br><br>HOCSA:<br>23% Juxtapositional Lobule Cortex (formerly Supplementary Cortex), 20% Superior Frontal Gyrus
9a	-19.372	11.803	67.25	-4.132		JHA:<br>16% GM Premotor cortex BA6 L<br><br>HOCSA:<br>42% Superior Frontal Gyrus, 1% Middle Frontal Gyrus
10	17.947	-97.668	-1.5	-4.662	629	JHA:<br>88% GM Visual cortex V1 BA17 R, 51% WM Optic radiation GM Visual cortex V2 BA18 R<br><br>HOCSA:<br>62% Occipital Pole, 1% Lateral Occipital Cortex, inferior divis
10a	15.459	-102.644	6.75	-4.081		JHA:<br>78% GM Visual cortex V1 BA17 R, 38% GM Visual cortex V R, 26% WM Optic radiation R<br><br>HOCSA:<br>71% Occipital Pole
11	60.243	9.315	12.25	-4.513	391	JHA:<br>53% GM Broca's area BA44 R, 6% GM Secondary somatose cortex / Parietal operculum OP4 R, 2% GM Broca's area BA<br><br>HOCSA:<br>38% Precentral Gyrus, 28% Inferior Frontal Gyrus, pars oper
12	-14.396	-75.276	-48.25	-4.459	680	Could not be labeled
13	25.411	1.851	31.5	-4.356	306	JHA:<br>3% WM Callosal body
14	-44.252	-42.932	-18.0	-4.328	340	HOCSA:<br>26% Temporal Fusiform Cortex, posterior division, 21% Infer Temporal Gyrus, posterior division, 10% Inferior Temporal G temporooccipital part, 6% Temporal Occipital Fusiform Corte
15	37.851	-87.716	-9.75	-4.191	272	JHA:<br>44% GM Visual cortex V3V R, 36% GM Visual cortex V4 R, GM Visual cortex V2 BA18 R<br><br>HOCSA:<br>51% Lateral Occipital Cortex, inferior division, 21% Occipital 2% Occipital Fusiform Gyrus
16	47.803	-18.052	-7.0	-4.105	306	JHA:<br>11% GM Insula Id1 R

						HOCSA:<br>47% Superior Temporal Gyrus, posterior division, 19% Middl<br>Temporal Gyrus, posterior division, 1% Middle Temporal Gyr<br>anterior division, 1% Superior Temporal Gyrus, anterior divisi
17	-61.668	1.851	26.0	-3.822	238	JHA:<br>48% GM Premotor cortex BA6 L, 19% GM Broca's area BA4<br>10% GM Primary somatosensory cortex BA3b L, 6% GM Pri<br>somatosensory cortex BA1 L<br><br>HOCSA:<br>52% Precentral Gyrus, 3% Postcentral Gyrus

Supplementary Table 4: Whole-brain clusters of the generalized Psychophysiological Interaction (gPPI) effect with left HPC used as a seed region. No clusters survived correction in the whole-brain analysis (two-sided t-test, FDR-corrected using a voxel-level threshold of $p<0.01$). The table lists clusters uncorrected for multiple comparisons, with statistical significance threshold defined at a single voxel level of $z_{\alpha}=1.96$. Table displays MNI coordinates (X, Y, Z), statistical T values and atlas labels of peak voxels of the clusters. Atlas labels are based on the Juelich Histological Atlas (JHA) and Harvard-Oxford Cortical Structural Atlas (HOCSA). The labels were generated using FSL. Atlases are listed only if the labels were found for a given atlas. The letters after the cluster ID indicates its subcluster.

Cluster	X	Y	Z	T Value	Cluster Size (	Atlas label
1	-29.324	49.123	-12.5	4.41	783	HOCSA:<br>69% Frontal Pole
2	-41.764	46.635	28.75	4.095	1651	HOCSA:<br>34% Frontal Pole
2a	-51.716	41.659	23.25	2.972		HOCSA:<br>1% Frontal Pole
3	10.483	-32.98	42.5	4.045	13890	JHA:<br>39% GM Superior parietal lobule 5Ci R, 18% GM Premotor cortex BA6 GM Superior parietal lobule 5M R, 1% GM Primary motor cortex BA4a<br><br>HOCSA:<br>60% Cingulate Gyrus, posterior division, 9% Precuneous Cortex, 3% Postcentral Gyrus, 3% Precentral Gyrus
3a	7.995	-32.98	28.75	3.938		JHA:<br>50% WM Callosal body<br><br>HOCSA:<br>35% Cingulate Gyrus, posterior division
3b	5.507	-37.956	53.5	3.876		JHA:<br>79% GM Superior parietal lobule 5M R, 32% GM Primary motor cortex R, 20% GM Superior parietal lobule 5Ci R, 11% WM Corticospinal tract<br><br>HOCSA:<br>29% Precuneous Cortex, 29% Postcentral Gyrus, 10% Precentral Gyru Cingulate Gyrus, posterior division
3c	-4.444	-52.884	67.25	3.716		JHA:<br>40% GM Superior parietal lobule 5L L, 32% GM Superior parietal lobul 22% GM Primary somatosensory cortex BA3b L, 14% GM Superior par lobule 7PC L, 11% GM Superior parietal lobule 5M L, 4% GM Primary r cortex BA4a L, 3% GM Primary motor cortex BA4p L<br><br>HOCSA:<br>41% Precuneous Cortex, 7% Postcentral Gyrus, 6% Superior Parietal L 2% Lateral Occipital Cortex, superior division
4	37.851	-23.028	-20.75	3.972	1072	JHA:<br>34% GM Hippocampus cornu ammonis R, 14% WM Optic radiation R, GM Hippocampus dentate gyrus R, 2% GM Hippocampus subiculum R<br><br>HOCSA:<br>38% Temporal Fusiform Cortex, posterior division, 5% Parahippocamp Gyrus, posterior division, 4% Parahippocampal Gyrus, anterior division Inferior Temporal Gyrus, posterior division, 1% Temporal Occipital Fusi Cortex
5	-14.396	-23.028	15.0	3.843	919	JHA:<br>1% WM Corticospinal tract L
5a	-16.884	-13.076	9.5	2.522		JHA:<br>37% WM Corticospinal tract L
6	-11.908	-40.444	83.75	3.795	1566	JHA:<br>4% GM Superior parietal lobule 5M L HOCSA: 2% Postcentral Gyrus

6a	-14.396	-52.884	78.25	3.556		JHA:<br>9% GM Superior parietal lobule 7PC L, 9% GM Superior parietal lobule [illegible]<br>6% GM Superior parietal lobule 5L L, 5% GM Primary motor cortex BA[illegible]<br>2% GM Superior parietal lobule 5M L<br><br>HOCSA:<br>9% Superior Parietal Lobule, 7% Postcentral Gyrus, 1% Lateral Occipit[illegible]<br>Cortex, superior division
6b	-11.908	-45.42	81.0	2.688		JHA:<br>41% GM Superior parietal lobule 5L L, 23% GM Superior parietal lobul[illegible]<br>19% GM Primary somatosensory cortex BA1 L, 11% GM Primary somatosensory cortex BA3b L, 11% GM Primary motor cortex BA4a L, [illegible]<br>Primary motor cortex BA4p L, 4% GM Premotor cortex BA6 L<br><br>HOCSA:<br>17% Postcentral Gyrus, 4% Superior Parietal Lobule
6c	-26.836	-40.444	72.75	2.436		JHA:<br>53% GM Primary somatosensory cortex BA1 L, 24% GM Primary somatosensory cortex BA2 L, 23% GM Superior parietal lobule 5L L, 1[illegible]<br>Superior parietal lobule 7PC L, 16% GM Superior parietal lobule 7A L, [illegible]<br>GM Primary motor cortex BA4a L, 8% GM Primary motor cortex BA4p [illegible]<br>GM Primary somatosensory cortex BA3b L, 1% WM Corticospinal tract [illegible]<br><br>HOCSA:<br>33% Postcentral Gyrus, 11% Superior Parietal Lobule, 3% Supramargi[illegible]<br>Gyrus, anterior division
7	0.531	19.267	34.25	3.756	7813	HOCSA:<br>58% Cingulate Gyrus, anterior division, 22% Paracingulate Gyrus
7a	10.483	-3.124	53.5	3.708		JHA:<br>45% GM Premotor cortex BA6 R<br><br>HOCSA:<br>34% Juxtapositional Lobule Cortex (formerly Supplementary Motor Co[illegible]<br>Precentral Gyrus, 1% Cingulate Gyrus, anterior division
7b	-9.42	-3.124	39.75	3.414		JHA:<br>1% WM Cingulum L, 1% GM Premotor cortex BA6 L<br><br>HOCSA:<br>22% Juxtapositional Lobule Cortex (formerly Supplementary Motor Co[illegible]<br>19% Cingulate Gyrus, anterior division
7c	3.019	1.851	48.0	3.175		JHA:<br>44% GM Premotor cortex BA6 R<br><br>HOCSA:<br>58% Juxtapositional Lobule Cortex (formerly Supplementary Motor Co[illegible]<br>24% Cingulate Gyrus, anterior division, 3% Paracingulate Gyrus
8	35.363	14.291	-1.5	3.75	408	JHA:<br>17% WM Inferior occipito-frontal fascicle R<br><br>HOCSA:<br>61% Insular Cortex
9	40.339	46.635	28.75	3.568	1889	HOCSA:<br>85% Frontal Pole, 1% Middle Frontal Gyrus
9a	27.899	59.075	28.75	2.523		HOCSA:<br>35% Frontal Pole
10	15.459	-8.1	4.0	3.55	2161	JHA:<br>25% WM Corticospinal tract R
10a	7.995	-10.588	15.0	3.32		JHA:<br>11% WM Fornix
10b	12.971	-20.54	12.25	2.31		JHA:<br>1% WM Fornix
11	65.219	-10.588	42.5	3.536	187	HOCSA:<br>2% Postcentral Gyrus
12	47.803	-77.764	37.0	3.504	1395	JHA:<br>3% GM Inferior parietal lobule PGp R<br><br>HOCSA:

						12% Lateral Occipital Cortex, superior division
12a	42.827	-65.324	28.75	2.887		JHA:<br>50% GM Inferior parietal lobule PGp R<br><br>HOCSA:<br>46% Lateral Occipital Cortex, superior division, 1% Angular Gyrus
12b	47.803	-75.276	26.0	2.432		JHA:<br>78% GM Inferior parietal lobule PGp R<br><br>HOCSA:<br>73% Lateral Occipital Cortex, superior division, 1% Lateral Occipital Co<br>inferior division
13	-36.788	4.339	17.75	3.493	766	JHA:<br>6% GM Broca's area BA44 L<br><br>HOCSA:<br>7% Central Opercular Cortex
14	52.779	-35.468	31.5	3.467	4528	JHA:<br>23% GM Inferior parietal lobule PFm R, 21% GM Inferior parietal lobule<br>20% GM Inferior parietal lobule PFcm R, 12% GM Anterior intra-parieta<br>hIP2 R, 8% GM Anterior intra-parietal sulcus hIP1 R<br><br>HOCSA:<br>16% Parietal Operculum Cortex, 15% Supramarginal Gyrus, posterior d<br>5% Supramarginal Gyrus, anterior division, 4% Planum Temporale, 1%<br>Angular Gyrus
14a	62.731	-30.492	28.75	3.251		JHA:<br>73% GM Inferior parietal lobule PF R, 45% GM Inferior parietal lobule F<br>3% GM Inferior parietal lobule PFop R, 2% GM Secondary somatosens<br>cortex / Parietal operculum OP1 R<br><br>HOCSA:<br>40% Supramarginal Gyrus, anterior division, 20% Parietal Operculum C<br>13% Planum Temporale, 4% Supramarginal Gyrus, posterior division, 1<br>Superior Temporal Gyrus, posterior division
14b	62.731	-40.444	31.5	2.987		JHA:<br>83% GM Inferior parietal lobule PF R, 50% GM Inferior parietal lobule F<br>1% GM Inferior parietal lobule Pga R<br><br>HOCSA:<br>58% Supramarginal Gyrus, posterior division, 10% Angular Gyrus, 4%<br>Temporale, 2% Supramarginal Gyrus, anterior division
14c	65.219	-50.396	34.25	2.859		HOCSA:<br>13% Angular Gyrus, 1% Supramarginal Gyrus, posterior divisio
15	35.363	26.73	-20.75	3.372	442	HOCSA:<br>75% Frontal Orbital Cortex
16	-71.62	-28	26.0	3.316	1225	No label found
16a	-61.668	-35.468	26.0	2.734		JHA:<br>80% GM Inferior parietal lobule PF L, 6% GM Inferior parietal lobule PF<br>4% GM Anterior intra-parietal sulcus hIP2 L, 3% GM Inferior parietal lol<br>PFm L<br><br>HOCSA:<br>41% Supramarginal Gyrus, anterior division, 21% Parietal Operculum C<br>13% Planum Temporale, 5% Supramarginal Gyrus, posterior division
17	-29.324	6.827	-29.0	3.241	323	HOCSA:<br>73% Temporal Pole, 3% Parahippocampal Gyrus, anterior division
18	-41.764	-32.98	-45.5	3.231	544	No label found
18a	-41.764	-45.42	-51.0	2.325		No label found
19	-11.908	-0.636	-1.5	3.185	510	No label found
20	-39.276	-18.052	28.75	3.137	561	JHA:<br>8% GM Primary somatosensory cortex BA3a L, 2% GM Secondary<br>somatosensory cortex / Parietal operculum OP3 L, 1% GM Insula Ig2 L<br>GM Secondary somatosensory cortex / Parietal operculum OP1 L<br><br>HOCSA:<br>2% Postcentral Gyrus

20a	-29.324	-20.54	31.5	2.623		JHA:<br>50% WM Corticospinal tract L, 23% WM Superior longitudinal fascicle L
20b	-31.812	-18.052	23.25	2.423		JHA:<br>25% WM Superior longitudinal fascicle L, 14% GM Secondary somatos cortex / Parietal operculum OP3 L, 11% GM Secondary somatosensory / Parietal operculum OP2 L, 9% WM Corticospinal tract L, 5% GM Insu<br><br>HOCSA:<br>1% Central Opercular Cortex
21	27.899	-23.028	-1.5	3.044	272	JHA:<br>67% WM Optic radiation R, 15% WM Acoustic radiation R, 2% GM Late geniculate body R, 1% WM Corticospinal tract R
22	37.851	-8.1	-4.25	3.032	1021	JHA:<br>12% WM Inferior occipito-frontal fascicle R, 8% GM Insula Id1 R, 2% W Acoustic radiation R, 1% GM Insula Ig2 R<br><br>HOCSA:<br>47% Insular Cortex
22a	37.851	-3.124	-15.25	2.153		JHA:<br>24% WM Optic radiation R, 23% WM Inferior occipito-frontal fascicle R, WM Uncinate fascicle R, 4% GM Insula Id1 R, 4% GM Amygdala_supe group R, 4% GM Amygdala_centromedial group R, 2% GM Amygdala_laterobasal group R<br><br>HOCSA:<br>13% Insular Cortex, 4% Planum Polare
23	-66.644	-37.956	42.5	3.017	714	HOCSA:<br>8% Supramarginal Gyrus, anterior division, 4% Supramarginal Gyrus, p division, 1% Planum Temporale, 1% Parietal Operculum Cortex
23a	-64.156	-30.492	48.0	2.633		HOCSA:<br>12% Supramarginal Gyrus, anterior division, 1% Postcentral Gyrus
24	47.803	19.267	-18.0	2.974	425	HOCSA:<br>67% Temporal Pole, 6% Frontal Orbital Cortex
25	10.483	-15.564	81.0	2.945	425	JHA:<br>51% GM Premotor cortex BA6 R, 6% GM Primary motor cortex BA4a R<br><br>HOCSA:<br>24% Precentral Gyrus, 4% Superior Frontal Gyrus
25a	17.947	-13.076	78.25	2.263		JHA:<br>98% GM Premotor cortex BA6 R<br><br>HOCSA:<br>24% Precentral Gyrus, 16% Superior Frontal Gyrus
26	55.267	16.779	-4.25	2.939	1174	JHA:<br>2% GM Broca's area BA45 R<br><br>HOCSA:<br>16% Temporal Pole, 11% Inferior Frontal Gyrus, pars opercularis, 2% F Operculum Cortex, 2% Frontal Orbital Cortex, 2% Precentral Gyrus, 2% Inferior Frontal Gyrus, pars triangularis, 1% Central Opercular Cortex
27	70.195	-13.076	26.0	2.926	493	JHA:<br>1% GM Secondary somatosensory cortex / Parietal operculum OP4 R, Secondary somatosensory cortex / Parietal operculum OP1 R, 1% GM parietal lobule PFt R, 1% GM Inferior parietal lobule PFop R, 1% GM In parietal lobule PF R<br><br>HOCSA:<br>4% Postcentral Gyrus, 1% Supramarginal Gyrus, anterior division
27a	67.707	-10.588	37.0	2.754		HOCSA:<br>1% Postcentral Gyrus
28	-11.908	-87.716	48.0	2.898	714	JHA:<br>11% GM Superior parietal lobule 7P L, 8% GM Superior parietal lobule<br><br>HOCSA:<br>10% Lateral Occipital Cortex, superior division, 9% Occipital Pole
29	-14.396	-0.636	75.5	2.888	476	JHA:<br>41% GM Premotor cortex BA6 L<br><br>HOCSA:

						22% Superior Frontal Gyrus
30	32.875	-0.636	28.75	2.859	578	JHA:<br>7% WM Corticospinal tract R
30a	25.411	-10.588	31.5	2.503		JHA:<br>42% WM Corticospinal tract R, 1% WM Superior occipito-frontal fascicl
31	12.971	9.315	4.0	2.849	459	No label found
32	0.531	-47.908	-73.0	2.82	425	No label found
33	-24.348	29.219	9.5	2.763	306	JHA:<br>12% WM Callosal body, 8% WM Superior occipito-frontal fascicle L<br><br>HOCSA:<br>1% Frontal Orbital Cortex
34	45.315	-23.028	64.5	2.735	255	JHA:<br>70% GM Primary somatosensory cortex BA1 R, 6% GM Premotor corte R, 6% GM Primary somatosensory cortex BA3b R, 4% GM Primary somatosensory cortex BA2 R, 4% GM Primary motor cortex BA4a R<br><br>HOCSA:<br>63% Postcentral Gyrus, 2% Precentral Gyrus
35	-16.884	31.707	-15.25	2.695	442	JHA:<br>3% WM Callosal body<br><br>HOCSA:<br>5% Frontal Orbital Cortex, 3% Frontal Pole, 2% Frontal Medial Cortex
35a	-14.396	21.755	-7.0	2.515		JHA:<br>27% WM Callosal body
36	-4.444	-35.468	-56.5	2.674	289	No label found
36a	-11.908	-37.956	-59.25	2.171		No label found
37	-24.348	6.827	26.0	2.654	340	JHA:<br>15% WM Superior occipito-frontal fascicle L
38	-31.812	31.707	-1.5	2.62	374	HOCSA:<br>31% Frontal Orbital Cortex, 3% Inferior Frontal Gyrus, pars triangularis Frontal Pole
39	-39.276	-13.076	-12.5	2.594	255	JHA:<br>20% WM Optic radiation L, 18% WM Inferior occipito-frontal fascicle L, Insula Id1 L<br><br>HOCSA:<br>18% Planum Polare, 1% Insular Cortex
40	-26.836	-97.668	-9.75	2.557	238	JHA:<br>47% GM Visual cortex V2 BA18 L, 35% GM Visual cortex V3V L, 22% Visual cortex V1 BA17 L, 7% WM Optic radiation L, 3% GM Visual corte<br><br>HOCSA:<br>62% Occipital Pole, 4% Lateral Occipital Cortex, inferior division
41	30.387	51.611	-12.5	2.546	510	HOCSA:<br>63% Frontal Pole
42	32.875	39.171	48.0	2.529	646	HOCSA:<br>15% Frontal Pole, 4% Middle Frontal Gyrus
42a	30.387	31.707	42.5	2.387		HOCSA:<br>48% Middle Frontal Gyrus, 10% Frontal Pole, 8% Superior Frontal Gyru
43	27.899	-95.18	-7.0	2.518	221	JHA:<br>76% GM Visual cortex V2 BA18 R, 43% GM Visual cortex V1 BA17 R, WM Optic radiation R, 20% GM Visual cortex V3V R<br><br>HOCSA:<br>58% Occipital Pole, 6% Lateral Occipital Cortex, inferior division
44	-4.444	-15.564	6.75	2.508	221	JHA:<br>1% WM Fornix
45	3.019	-3.124	26.0	2.507	527	JHA:

						88% WM Callosal body, 3% WM Cingulum R<br><br>HOCSA:<br>9% Cingulate Gyrus, anterior division, 1% Cingulate Gyrus, posterior di
45a	0.531	4.339	28.75	2.347		JHA:<br>28% WM Callosal body<br><br>HOCSA:<br>54% Cingulate Gyrus, anterior division
46	0.531	36.683	17.75	2.46	255	HOCSA:<br>66% Cingulate Gyrus, anterior division, 10% Paracingulate Gyrus
47	17.947	41.659	-15.25	2.429	170	HOCSA:<br>44% Frontal Pole, 2% Frontal Orbital Cortex
48	27.899	26.731	1.25	2.408	289	JHA:<br>2% WM Inferior occipito-frontal fascicle R<br><br>HOCSA:<br>10% Insular Cortex, 5% Frontal Orbital Cortex
49	57.755	-18.052	50.75	2.394	221	JHA:<br>74% GM Primary somatosensory cortex BA1 R, 38% GM Primary somatosensory cortex BA2 R, 18% GM Inferior parietal lobule PFt R, 2 Primary somatosensory cortex BA3b R<br><br>HOCSA:<br>55% Postcentral Gyrus, 10% Supramarginal Gyrus, anterior division
50	7.995	-77.764	39.75	2.386	221	JHA:<br>30% GM Superior parietal lobule 7M R, 28% GM Superior parietal lobu<br><br>HOCSA:<br>37% Precuneous Cortex, 28% Cuneal Cortex, 1% Lateral Occipital Cor superior division
51	17.947	-50.396	23.25	2.38	187	JHA:<br>49% WM Callosal body, 2% WM Optic radiation R<br><br>HOCSA:<br>12% Precuneous Cortex

Supplementary Table 5: Whole-brain clusters of the generalized Psychophysiological Interaction (gPPI) effect with SMA used as a seed region. No clusters survived correction in the whole-brain analysis (two-sided t-test, FDR-corrected using a voxel-level threshold of $p<0.01$). The table lists clusters uncorrected for multiple comparisons, with statistical significance threshold defined at a single voxel level of $z_{\alpha}=1.96$. Table displays MNI coordinates (X, Y, Z), statistical T values and atlas labels of peak voxels of the clusters. Atlas labels are based on the Juelich Histological Atlas (JHA) and Harvard-Oxford Cortical Structural Atlas (HOCSA). The labels were generated using FSL. Atlases are listed only if the labels were found for a given atlas. The letters after the cluster ID indicates its subcluster.

Cluster	X	Y	Z	T Value	Cluster Size ([illegible]	Atlas label
1	32.875	-40.444	-9.75	4.459	1855	JHA:<br>23% WM Optic radiation R, 7% GM Hippocampus cornu ammonis R[illegible] WM Callosal body, 4% GM Hippocampus subiculum R, 4% GM Hippocampus dentate gyrus R<br><br>HOCSA:<br>38% Lingual Gyrus, 23% Temporal Occipital Fusiform Cortex, 12% Parahippocampal Gyrus, posterior division, 9% Temporal Fusiform C[illegible] posterior division
1a	22.923	-45.42	-9.75	2.66		JHA:<br>1% GM Visual cortex V2 BA18 R<br><br>HOCSA:<br>68% Lingual Gyrus, 14% Temporal Occipital Fusiform Cortex, 1% Te[illegible] Fusiform Cortex, posterior division
1b	27.899	-55.372	-7.0	2.057		JHA:<br>5% WM Optic radiation R, 1% GM Visual cortex V2 BA18 R<br><br>HOCSA:<br>37% Temporal Occipital Fusiform Cortex, 27% Lingual Gyrus, 6% Oc[illegible] Fusiform Gyrus
2	-49.228	-5.612	45.25	4.247	5940	JHA:<br>79% GM Premotor cortex BA6 L, 23% GM Primary motor cortex BA4[illegible] WM Corticospinal tract L, 7% GM Primary somatosensory cortex BA[illegible]<br><br>HOCSA:<br>57% Precentral Gyrus, 3% Postcentral Gyrus
2a	-49.228	-5.612	53.5	3.663		JHA:<br>45% GM Premotor cortex BA6 L, 4% GM Primary motor cortex BA4a[illegible] GM Primary somatosensory cortex BA1 L<br><br>HOCSA:<br>55% Precentral Gyrus, 2% Middle Frontal Gyrus, 1% Postcentral Gy[illegible]
2b	-56.692	-5.612	48.0	3.541		JHA:<br>39% GM Premotor cortex BA6 L, 9% GM Primary motor cortex BA4a[illegible] GM Primary somatosensory cortex BA1 L<br><br>HOCSA:<br>46% Precentral Gyrus, 2% Postcentral Gyrus
2c	-61.668	-13.076	26.0	3.208		JHA:<br>50% GM Secondary somatosensory cortex / Parietal operculum OP4[illegible] GM Primary somatosensory cortex BA1 L, 22% GM Inferior parietal l[illegible] PFop L, 16% GM Inferior parietal lobule PFt L, 10% GM Secondary somatosensory cortex / Parietal operculum OP1 L, 10% GM Primary somatosensory cortex BA3b L, 10% GM Primary somatosensory cor[illegible] L, 8% GM Primary somatosensory cortex BA3a L, 1% GM Broca's ar[illegible] BA44 L<br><br>HOCSA:<br>74% Postcentral Gyrus, 3% Supramarginal Gyrus, anterior division
3	15.459	6.827	37.0	3.912	1293	JHA:<br>29% WM Callosal body
4	-66.644	-13.076	-1.5	3.696	6655	HOCSA:

						45% Superior Temporal Gyrus, posterior division, 6% Middle Tempor<br>Gyrus, anterior division, 5% Middle Temporal Gyrus, posterior divisio<br>Superior Temporal Gyrus, anterior division, 1% Planum Temporale
4a	-59.18	9.315	4.0	3.667		JHA:<br>38% GM Broca's area BA44 L, 10% GM Broca's area BA45 L<br><br>HOCSA:<br>20% Inferior Frontal Gyrus, pars opercularis, 17% Precentral Gyrus
4b	-49.228	9.315	12.25	3.524		JHA:<br>35% GM Broca's area BA44 L, 2% GM Broca's area BA45 L<br><br>HOCSA:<br>40% Inferior Frontal Gyrus, pars opercularis, 10% Precentral Gyrus
4c	-61.668	-25.516	4.0	2.657		JHA:<br>10% GM Secondary somatosensory cortex / Parietal operculum OP1<br>GM Primary auditory cortex TE1.0 L<br><br>HOCSA:<br>40% Superior Temporal Gyrus, posterior division, 14% Planum Temp<br>3% Middle Temporal Gyrus, posterior division
5	-31.812	-18.052	23.25	3.608	1123	JHA:<br>25% WM Superior longitudinal fascicle L, 14% GM Secondary<br>somatosensory cortex / Parietal operculum OP3 L, 11% GM Seconda<br>somatosensory cortex / Parietal operculum OP2 L, 9% WM Corticosp<br>tract L, 5% GM Insula Ig2 L<br><br>HOCSA:<br>1% Central Opercular Cortex
5a	-26.836	-10.588	31.5	3.231		JHA:<br>62% WM Corticospinal tract L, 3% WM Superior occipito-frontal fasci
6	-16.884	-95.18	-23.5	3.608	1174	HOCSA:<br>10% Occipital Pole, 3% Occipital Fusiform Gyrus
6a	-16.884	-92.692	-34.5	2.529		No label found
6b	-31.812	-92.692	-20.75	2.515		JHA:<br>11% GM Visual cortex V3V L, 7% GM Visual cortex V4 L, 3% GM Vis<br>cortex V2 BA18 L<br><br>HOCSA:<br>14% Lateral Occipital Cortex, inferior division, 11% Occipital Pole, 3%<br>Occipital Fusiform Gyrus
6c	-9.42	-100.156	-12.5	2.231		JHA:<br>71% GM Visual cortex V1 BA17 L, 38% WM Optic radiation L, 37% G<br>Visual cortex V2 BA18 L, 5% GM Visual cortex V3V L<br><br>HOCSA:<br>52% Occipital Pole, 2% Lateral Occipital Cortex, inferior division
7	62.731	-18.052	-1.5	3.597	2996	HOCSA:<br>49% Superior Temporal Gyrus, posterior division, 11% Middle Tempo<br>Gyrus, posterior division, 2% Planum Temporale, 1% Superior Temp<br>Gyrus, anterior division
7a	70.195	-13.076	-1.5	3.519		HOCSA:<br>30% Superior Temporal Gyrus, posterior division, 3% Middle Tempor<br>Gyrus, posterior division
7b	52.779	-20.54	-4.25	3.399		JHA:<br>1% GM Insula Id1 R<br><br>HOCSA:<br>53% Superior Temporal Gyrus, posterior division, 14% Middle Tempo<br>Gyrus, posterior division
7c	70.195	-8.1	-1.5	2.996		HOCSA:<br>9% Superior Temporal Gyrus, posterior division, 2% Superior Tempo<br>Gyrus, anterior division
8	-1.956	1.851	67.25	3.536	2212	JHA:<br>74% GM Premotor cortex BA6 L<br><br>HOCSA:<br>72% Juxtapositional Lobule Cortex (formerly Supplementary Motor C<br>3% Superior Frontal Gyrus

9	-19.372	-42.932	-4.25	3.449	3455	JHA:<br>22% GM Hippocampus subiculum L, 19% WM Callosal body, 12% W<br>Cingulum L, 2% WM Optic radiation L, 2% GM Hippocampus cornu a<br>L<br><br>HOCSA:<br>8% Lingual Gyrus, 7% Parahippocampal Gyrus, posterior division, 6%<br>Cingulate Gyrus, posterior division
9a	-21.86	-35.468	1.25	3.205		JHA:<br>63% GM Hippocampus dentate gyrus L, 50% WM Fornix, 47% GM<br>Hippocampus cornu ammonis L, 4% WM Callosal body, 1% GM<br>Hippocampus subiculum L
9b	-29.324	-37.956	-7.0	3.151		JHA:<br>78% GM Hippocampus cornu ammonis L, 28% GM Hippocampus de<br>gyrus L, 15% WM Optic radiation L, 7% WM Callosal body, 4% WM F<br><br>HOCSA:<br>18% Parahippocampal Gyrus, posterior division, 4% Lingual Gyrus, 2<br>Temporal Fusiform Cortex, posterior division, 1% Temporal Occipital<br>Fusiform Cortex
9c	-29.324	-50.396	-7.0	3.09		JHA:<br>4% WM Optic radiation L<br><br>HOCSA:<br>54% Temporal Occipital Fusiform Cortex, 17% Lingual Gyrus, 5% Te<br>Fusiform Cortex, posterior division
10	7.995	-13.076	-37.25	3.405	170	No label found
11	45.315	11.803	-31.75	3.385	1719	HOCSA:<br>60% Temporal Pole
11a	45.315	21.755	-31.75	3.226		HOCSA:<br>53% Temporal Pole
12	-21.86	-35.468	-23.5	3.253	374	HOCSA:<br>7% Parahippocampal Gyrus, posterior division, 6% Temporal Fusifor<br>Cortex, posterior division
13	-4.444	46.635	56.25	3.168	783	HOCSA:<br>1% Frontal Pole
13a	3.019	46.635	50.75	2.662		HOCSA:<br>20% Frontal Pole, 18% Superior Frontal Gyrus
14	65.219	-10.588	28.75	3.086	1872	JHA:<br>28% GM Primary somatosensory cortex BA1 R, 24% GM Secondary<br>somatosensory cortex / Parietal operculum OP4 R, 15% GM Primary<br>somatosensory cortex BA3b R, 13% GM Primary somatosensory cor<br>R, 10% GM Inferior parietal lobule PFt R, 8% GM Secondary somato<br>cortex / Parietal operculum OP1 R, 7% GM Inferior parietal lobule PF<br>4% GM Primary motor cortex BA4p R, 2% GM Inferior parietal lobule<br>1% GM Primary motor cortex BA4a R<br><br>HOCSA:<br>74% Postcentral Gyrus, 1% Precentral Gyrus
14a	55.267	-8.1	53.5	2.963		JHA:<br>14% GM Premotor cortex BA6 R, 4% GM Primary somatosensory co<br>BA1 R, 2% GM Primary somatosensory cortex BA3b R<br><br>HOCSA:<br>5% Precentral Gyrus, 4% Postcentral Gyrus
14b	57.755	1.851	45.25	2.124		JHA:<br>10% GM Premotor cortex BA6 R, 1% GM Primary somatosensory co<br>BA3b R<br><br>HOCSA:<br>21% Precentral Gyrus, 1% Middle Frontal Gyrus
15	-24.348	-57.86	1.25	3.083	1072	JHA:<br>41% GM Visual cortex V2 BA18 L, 40% WM Optic radiation L, 39% G<br>Visual cortex V1 BA17 L, 22% WM Callosal body, 2% GM Visual cort<br><br>HOCSA:<br>15% Lingual Gyrus, 7% Precuneous Cortex, 3% Intracalcarine Cortex<br>Cingulate Gyrus, posterior division
15a	-26.836	-67.812	-7.0	2.259		JHA:

						31% GM Visual cortex V4 L<br><br>HOCSA:<br>46% Occipital Fusiform Gyrus, 9% Temporal Occipital Fusiform Corte<br>Lingual Gyrus
16	-26.836	21.755	-26.25	3.049	323	HOCSA:<br>38% Frontal Orbital Cortex, 1% Temporal Pole
16a	-21.86	14.291	-23.5	2.566		HOCSA:<br>78% Frontal Orbital Cortex
17	-36.788	16.779	-4.25	3.042	1702	HOCSA:<br>64% Insular Cortex, 2% Central Opercular Cortex, 1% Frontal Operc<br>Cortex
17a	-39.276	24.243	-7.0	2.992		HOCSA:<br>70% Frontal Orbital Cortex, 4% Frontal Operculum Cortex
17b	-36.788	11.803	1.25	2.697		HOCSA:<br>58% Insular Cortex, 7% Frontal Operculum Cortex, 5% Central Oper<br>Cortex
17c	-34.3	11.803	12.25	2.114		JHA:<br>8% GM Broca's area BA44 L<br><br>HOCSA:<br>44% Frontal Operculum Cortex, 14% Central Opercular Cortex, 8% I<br>Cortex
18	-21.86	11.803	42.5	2.986	408	HOCSA:<br>7% Superior Frontal Gyrus, 1% Middle Frontal Gyrus
19	-44.252	-28.004	28.75	2.964	221	JHA:<br>9% GM Secondary somatosensory cortex / Parietal operculum OP1 L<br>GM Inferior parietal lobule PFop L, 2% GM Inferior parietal lobule PF<br><br>HOCSA:<br>3% Supramarginal Gyrus, anterior division, 2% Parietal Operculum C<br>2% Central Opercular Cortex
20	0.531	-57.86	-23.5	2.872	1123	No label found
20a	15.459	-62.836	-23.5	2.529		No label found
20b	0.531	-57.86	-34.5	2.28		No label found
21	40.339	16.779	-4.25	2.848	885	JHA:<br>4% WM Inferior occipito-frontal fascicle R<br><br>HOCSA:<br>73% Insular Cortex, 2% Frontal Operculum Cortex, 2% Frontal Orbita<br>Cortex
22	-64.156	9.315	23.25	2.842	170	JHA:<br>15% GM Broca's area BA44 L, 2% GM Premotor cortex BA6 L, 1% G<br>Broca's area BA45 L<br><br>HOCSA:<br>3% Precentral Gyrus
23	-9.42	-10.588	37.0	2.842	697	JHA:<br>26% WM Cingulum L, 21% WM Callosal body<br><br>HOCSA:<br>11% Cingulate Gyrus, anterior division, 1% Cingulate Gyrus, posterio<br>division, 1% Juxtapositional Lobule Cortex (formerly Supplementary I<br>Cortex)
23a	-14.396	-18.052	45.25	2.25		JHA:<br>22% WM Corticospinal tract L, 11% GM Premotor cortex BA6 L, 9%<br>Superior parietal lobule 5Ci L<br><br>HOCSA:<br>8% Precentral Gyrus, 2% Cingulate Gyrus, posterior division, 1% Cin<br>Gyrus, anterior division, 1% Juxtapositional Lobule Cortex (formerly<br>Supplementary Motor Cortex)
24	-51.716	-37.956	-31.75	2.829	493	HOCSA:

						5% Inferior Temporal Gyrus, posterior division, 1% Inferior Temporal temporooccipital part
24a	-51.716	-37.956	-42.75	2.304		No label found
25	-29.324	66.539	20.5	2.818	340	HOCSA:<br>3% Frontal Pole
25a	-34.3	59.075	26.0	2.694		HOCSA:<br>2% Frontal Pole
26	-54.204	-77.764	-4.25	2.815	340	HOCSA:<br>25% Lateral Occipital Cortex, inferior division
26a	-49.228	-85.228	-4.25	2.692		HOCSA:<br>13% Lateral Occipital Cortex, inferior division
27	10.483	24.243	6.75	2.813	425	JHA:<br>53% WM Callosal body
28	-26.836	-15.564	-18.0	2.802	817	JHA:<br>88% GM Hippocampus cornu ammonis L, 58% GM Hippocampus de gyrus L, 31% GM Hippocampus subiculum L, 17% GM Amygdala_laterobasal group L, 14% GM Amygdala_superficial group WM Fornix, 2% GM Hippocampus hippocampal-amygdaloid transitio
28a	-34.3	-23.028	-20.75	2.516		JHA:<br>68% GM Hippocampus cornu ammonis L, 26% GM Hippocampus de gyrus L, 14% WM Optic radiation L, 12% GM Hippocampus subiculu WM Fornix<br><br>HOCSA:<br>21% Temporal Fusiform Cortex, posterior division, 17% Parahippoca Gyrus, posterior division, 15% Parahippocampal Gyrus, anterior divis
29	-1.956	-35.468	-59.25	2.784	238	No label found
30	0.531	44.147	-1.5	2.747	272	HOCSA:<br>44% Cingulate Gyrus, anterior division, 42% Paracingulate Gyrus, 2% Frontal Medial Cortex
31	-39.276	64.051	-9.75	2.705	170	HOCSA:<br>4% Frontal Pole
32	25.411	-85.228	31.5	2.698	306	HOCSA:<br>48% Lateral Occipital Cortex, superior division, 24% Occipital Pole
33	-29.324	-23.028	56.25	2.69	187	JHA:<br>61% WM Corticospinal tract L, 36% GM Primary motor cortex BA4p L GM Primary motor cortex BA4a L, 15% GM Premotor cortex BA6 L, 8 Primary somatosensory cortex BA3b L, 1% GM Primary somatosens cortex BA3a L, 1% GM Primary somatosensory cortex BA1 L<br><br>HOCSA:<br>31% Precentral Gyrus, 17% Postcentral Gyrus
34	32.875	11.803	53.5	2.684	187	HOCSA:<br>34% Middle Frontal Gyrus, 5% Superior Frontal Gyrus, 1% Precentra
35	7.995	-40.444	-34.5	2.641	204	No label found
36	-19.372	69.027	9.5	2.614	595	HOCSA:<br>41% Frontal Pole
36a	-29.324	66.539	4.0	2.534		HOCSA:<br>45% Frontal Pole
37	57.755	-35.468	56.25	2.614	663	HOCSA:<br>16% Supramarginal Gyrus, posterior division, 1% Supramarginal Gyr anterior division
38	3.019	14.291	12.25	2.508	510	JHA:<br>12% WM Callosal body
39	-6.932	-23.028	67.25	2.443	272	JHA:

						60% GM Premotor cortex BA6 L, 50% GM Primary motor cortex BA4<br>39% WM Corticospinal tract L<br><br>HOCSA:<br>33% Precentral Gyrus
39a	-11.908	-15.564	67.25	2.401		JHA:<br>89% GM Premotor cortex BA6 L, 12% GM Primary motor cortex BA4<br>11% WM Corticospinal tract L<br><br>HOCSA:<br>23% Precentral Gyrus, 5% Superior Frontal Gyrus, 1% Juxtapositiona<br>Lobule Cortex (formerly Supplementary Motor Cortex)
40	37.851	34.195	-23.5	2.416	204	HOCSA:<br>4% Frontal Orbital Cortex, 2% Frontal Pole
41	-34.3	-3.124	-1.5	2.41	204	JHA:<br>7% WM Inferior occipito-frontal fascicle L<br><br>HOCSA:<br>1% Insular Cortex
42	-11.908	-37.956	70.0	2.361	204	JHA:<br>38% GM Primary motor cortex BA4a L, 26% WM Corticospinal tract L<br>GM Primary somatosensory cortex BA3b L, 16% GM Primary motor c<br>BA4p L, 14% GM Superior parietal lobule 5L L, 13% GM Premotor co<br>BA6 L, 10% GM Primary somatosensory cortex BA3a L, 9% GM Sup<br>parietal lobule 5M L, 9% GM Primary somatosensory cortex BA1 L, 8<br>Primary somatosensory cortex BA2 L<br><br>HOCSA:<br>43% Postcentral Gyrus, 9% Precentral Gyrus, 2% Superior Parietal L
43	-29.324	-85.228	31.5	2.28	255	JHA:<br>30% GM Inferior parietal lobule PGp L<br><br>HOCSA:<br>64% Lateral Occipital Cortex, superior division, 9% Occipital Pole
