# Supplementary material for: Hippocampal-entorhinal cognitive maps and cortical motor system represent action plans and their outcomes
Source: Nat Commun. 2025 May 3;16:4139. doi: 10.1038/s41467-025-59153-y (PMC12049502; doi:10.1038/s41467-025-59153-y)
Supplement: Supplementary file 2 — Description of Additional Supplementary Files [file 41467_2025_59153_MOESM2_ESM.pdf]

Supplementary Movie 1: Guided Exploration Task. The movie demonstrates a trial of the Guided Exploration Task. In this task, participants were taught the mapping between actions and their outcomes. They were presented with two joysticks and arrows indicating which actions to perform with each joystick. Participants moved the joysticks to one of five positions. Once a valid combination of actions had been performed, the catapult was activated by touching a virtual button. It launched 10 balls, pausing after 5 throws to allow participants to repeat the initial actions with the joysticks. Different actions were associated with different probabilities of catching the ball (joystick 1 on the left) and probabilities of the ball remaining visible for its entire trajectory (joystick 2 on the right), which the participants were instructed to find out and remember. A transparent yellow square appeared at the initial position of each joystick after the action was performed. The purpose of this square was to guide participants to return the joystick to its upright position, allowing them to perform either a different action or the same action with greater precision, if necessary.

Supplementary Movie 2: Goal-directed Action Task. The movie demonstrates a pair of trials of the Goal-directed Action Task, testing participants' knowledge of action-outcome associations and their relations to each other. In the first trial of the pair, participants were instructed to produce one of the coloured balls, here the purple ball. The second subsequent trial instructed participants to use the purple ball as a reference point and to select a correct combination of actions from the cued alternatives following the instructions on the screen. The screen here is showing the following instructions: "From the displayed actions, select a combination that will allow you to catch the ball with a lower probability and let the ball remain visible with a higher probability compared to the purple ball". A transparent yellow square appeared at the initial position of each joystick after the action was performed. The purpose of this square was to guide participants to return the joystick to its upright position, allowing them to perform either a different action or the same action with greater precision, if necessary.
