# Supplementary material for: Hippocampal-entorhinal cognitive maps and cortical motor system represent action plans and their outcomes
Source: Nat Commun. 2025 May 3;16:4139. doi: 10.1038/s41467-025-59153-y (PMC12049502; doi:10.1038/s41467-025-59153-y)
Supplement: Supplementary file 3 — Reporting Summary [file 41467_2025_59153_MOESM3_ESM.pdf]

nature portfolio

Corresponding author(s): Irina Barnaveli
barnaveli@cbs.mpg.de
Christian F. Doeller
doeller@cbs.mpg.de

Last updated by author(s): 2025.04.05

Reporting Summary

Nature Portfolio wishes to improve the reproducibility of the work that we publish. This form provides structure for consistency and transparency in reporting. For further information on Nature Portfolio policies, see our Editorial Policies and the Editorial Policy Checklist.

Statistics

For all statistical analyses, confirm that the following items are present in the figure legend, table legend, main text, or Methods section.

n/a	Confirmed	
☐	☒	The exact sample size (n) for each experimental group/condition, given as a discrete number and unit of measurement
☐	☒	A statement on whether measurements were taken from distinct samples or whether the same sample was measured repeatedly
☐	☒	The statistical test(s) used AND whether they are one- or two-sided<br>*Only common tests should be described solely by name; describe more complex techniques in the Methods section.*
☐	☒	A description of all covariates tested
☐	☒	A description of any assumptions or corrections, such as tests of normality and adjustment for multiple comparisons
☐	☒	A full description of the statistical parameters including central tendency (e.g. means) or other basic estimates (e.g. regression coefficient) AND variation (e.g. standard deviation) or associated estimates of uncertainty (e.g. confidence intervals)
☐	☒	For null hypothesis testing, the test statistic (e.g. F, t, r) with confidence intervals, effect sizes, degrees of freedom and P value noted<br>*Give P values as exact values whenever suitable.*
☒	☐	For Bayesian analysis, information on the choice of priors and Markov chain Monte Carlo settings
☐	☒	For hierarchical and complex designs, identification of the appropriate level for tests and full reporting of outcomes
☐	☒	Estimates of effect sizes (e.g. Cohen's d, Pearson's r), indicating how they were calculated

Our web collection on statistics for biologists contains articles on many of the points above.

Software and code

Policy information about availability of computer code

Data collection	The immersive virtual reality (VR) as well as the computer tasks were created and presented using a cross-platform game engine developed by Unity Technologies (version 2018.4.30f1, https://unity.com/).<br>MRI data were acquired using a 32-channel head coil on a 3 Tesla Siemens Magnetom SkyraFit system (Siemens, Erlangen, Germany). Task stimuli were presented to participants on a screen, which they viewed through a mirror fixed to the head coil.
Data analysis	MRI analyses were carried out using the tools dcm2bids (version 2.1.6, https://unfmontreal.github.io/Dcm2Bids/), fMRIPrep 21.0.2 (Esteban, Markiewicz, et al. (2018); Esteban, Blair, et al. (2018); RRID:SCR_016216), Python 3.8 using Spyder (https://www.spyder-ide.org/) distributed via Anaconda (https://www.anaconda.com/), RSA Toolbox (rsatoolbox, version 0.1.2, https://rsatoolbox.readthedocs.io/en/stable/), CONN toolbox (Whitfield-Gabrieli & Nieto-Castanon (2012); RRID:SCR_009550).<br>Behavioural analyses were carried out using MATLAB 2019b and R 4.2.2.

For manuscripts utilizing custom algorithms or software that are central to the research but not yet described in published literature, software must be made available to editors and reviewers. We strongly encourage code deposition in a community repository (e.g. GitHub). See the Nature Portfolio guidelines for submitting code & software for further information.

Data

Policy information about availability of data

All manuscripts must include a data availability statement. This statement should provide the following information, where applicable:

- Accession codes, unique identifiers, or web links for publicly available datasets
- A description of any restrictions on data availability
- For clinical datasets or third party data, please ensure that the statement adheres to our policy

Raw data are protected and are not available due to data privacy. Preprocessed data will be made available upon request to the corresponding author. The processed data to reproduce the statistical analyses reported in this paper are available on the Open Science Framework https://doi.org/10.17605/OSF.IO/UZ83D. Source data are provided as a Source Data file.

Research involving human participants, their data, or biological material

Policy information about studies with human participants or human data. See also policy information about sex, gender (identity/presentation), and sexual orientation and race, ethnicity and racism.

Reporting on sex and gender	46 participants in total, 22 females, as indicated by the participants themselves. We gathered data from both sexes but did not evaluate gender differences, as we lacked a hypothesis regarding how cognitive maps might vary across the gender spectrum
Reporting on race, ethnicity, or other socially relevant groupings	No socially relevant groupings were considered in the study.
Population characteristics	Age range of participants was 19-35 years, mean age 26.6 years, standard deviation 4.8 years. All participants had normal or corrected-to-normal vision, no history of or current neurological or psychiatric disorders, were right-handed and met MRI safety criteria.
Recruitment	Participants were recruited using the participant database of the Max Planck Institute for Human Cognitive and Brain Sciences, Leipzig, Germany.
Ethics oversight	The study was approved by the local Ethics Committee of Leipzig University, Germany (protocol number 112/21-ek).

Note that full information on the approval of the study protocol must also be provided in the manuscript.

Field-specific reporting

Please select the one below that is the best fit for your research. If you are not sure, read the appropriate sections before making your selection.

[x] Life sciences [ ] Behavioural & social sciences [ ] Ecological, evolutionary & environmental sciences

For a reference copy of the document with all sections, see nature.com/documents/nr-reporting-summary-flat.pdf

Life sciences study design

All studies must disclose on these points even when the disclosure is negative.

Sample size	Sample size was determined without an priori power analysis, as there are no studies examining the emergence of hippocampal-entorhinal abstract map-like representations in motor action planning and comparison. The sample size was chosen on the basis of experiments investigating hippocampal-entorhinal map-like representations in different domains, which have obtained meaningful results with smaller pools of participants.
Data exclusions	52 participants took part in the study. Four participants were excluded from the analysis due to technical problems with immersive VR. Additional two participants were excluded due to signal dropout in the entorhinal cortex. In total, 46 participants entered the analysis.
Replication	No replication was attempted because of the resources needed for running this fMRI study. The main effects are present in the majority of participants.
Randomization	Only one group of participants was tested and thus participants were not assigned to experimental groups.
Blinding	No group assignment took place, blinding was therefore not necessary.

Reporting for specific materials, systems and methods

We require information from authors about some types of materials, experimental systems and methods used in many studies. Here, indicate whether each material, system or method listed is relevant to your study. If you are not sure if a list item applies to your research, read the appropriate section before selecting a response.

Materials & experimental systems

n/a	Involved in the study
☒	☐ Antibodies
☒	☐ Eukaryotic cell lines
☒	☐ Palaeontology and archaeology
☒	☐ Animals and other organisms
☒	☐ Clinical data
☒	☐ Dual use research of concern
☒	☐ Plants

Methods

n/a	Involved in the study
☒	☐ ChIP-seq
☒	☐ Flow cytometry
☐	☒ MRI-based neuroimaging

Plants

Seed stocks	NA
Novel plant genotypes	NA
Authentication	NA

Magnetic resonance imaging

Experimental design

Design type	Task fMRI with block design
Design specifications	The Comparison Task from the first scanning session was divided into 6 runs of 24 trials each (a total of 144 trials). Each trial sequentially presented a pair of action combinations, each combination consisting of two images cueing one of the possible actions. Each combination was displayed on the screen for 3s, followed by a 1.5s inter-stimulus interval that included a fixation cross before the presentation of the next combination. The subsequent 4.5s reflection period with a displayed fixation cross was succeeded by a question or a statement (self-paced, max. 4.5s) and an inter-trial interval after that, the duration of which depended on the time participants took for their choice using a button box (choice + inter-trial interval always lasted a total of 7.5s).<br><br>The Comparison Task from the second scanning session consisted of 5 runs of 30 trials each (a total of 150 trials). Each trial sequentially presented a pair of coloured balls, each ball displayed on the screen for 1.5s with a 1.5s inter-stimulus interval in between. After a 4.5s reflection period, participants were shown a question or statement (self-paced, max. 4.5s) and an inter-trial interval after that (choice + inter-trial interval always lasted a total of 7.5s).
Behavioral performance measures	In the scanner, behavioural responses (button presses, response times) were collected with an MRI-compatible button box. In both tasks, the behavioural performance was assessed as the proportion of correctly answered trials.

Acquisition

Imaging type(s)	functional and structural MRI, fieldmap
Field strength	3T
Sequence & imaging parameters	MRI data were acquired using a 32-channel head coil on a 3 Tesla Siemens Magnetom SkyraFit system (Siemens, Erlangen, Germany). After a localizer scan, functional scans (fMRI) were acquired using T2*-weighted whole-brain gradient-echo echo planar imaging (GE-EPI) with multiband acceleration, sensitive to blood-oxygen-level-dependent (BOLD) contrast (Feinberg et al., 2010; Moeller et al., 2010). Settings of the fMRI sequence were as follows: TR = 1500 ms, TE = 22 ms, voxel size = 2.5 mm isotropic, FOV = 204 mm, flip angle = 80°, bandwidth = 1794 Hz/Px, 63 interleaved slices, distance factor = 10 %, phase encoding direction = A-P.<br><br>Between the task runs, field maps were acquired using the opposite phase-encoded EPIs with the following parameters: TR = 8000 ms; TE = 50 ms; voxel size = 2.5 mm isotropic; field of view = 204 mm; flip angle = 90°; partial fourier = 0.75; bandwidth = 1794 Hz/Px; multi-band acceleration factor = 1; 69 slices interleaved; slice thickness = 2.5 mm; distance factor = 0 %.

A T1-weighted MP2RAGE anatomical scan was obtained using following parameters: TR = 2300 ms; TE = 2.98 ms; voxel size = 1 mm isotropic; field of view = 256 mm; flip angle = 9°; bandwidth = 240 Hz/Px; slice thickness = 1 mm; distance factor = 50 %).

Area of acquisition: whole-brain

Diffusion MRI ☐ Used ☒ Not used

Preprocessing

Preprocessing software: DICOM files of the MRI scanner were converted to NIfTI files and reorganized according to the BIDS standard (K. J. Gorgolewski et al., 2016) using the tool dcm2bids (version 2.1.6, https://unfmontreal.github.io/Dcm2Bids/). The preprocessing was performed using fMRIPrep 21.0.2 (Esteban, Markiewicz, et al. (2018); Esteban, Blair, et al. (2018); RRID:SCR_016216), which is based on Nipype 1.6.1 (K. Gorgolewski et al. (2011); K. J. Gorgolewski et al. (2018); RRID:SCR_002502).

Normalization: The BOLD timeseries were resampled into standard space, generating a preprocessed BOLD run in MNI152NLin2009cAsym space.
The T1-weighted (T1w) image was corrected for intensity non-uniformity (INU) with N4BiasFieldCorrection (Tustison et al. 2010), distributed with ANTs 2.3.3 (Avants et al. 2008, RRID:SCR_004757), and used as T1w-reference throughout the workflow. The T1w-reference was then skull-stripped with a Nipype implementation of the antsBrainExtraction.sh workflow (from ANTs), using OASIS30ANTs as target template. Brain tissue segmentation of cerebrospinal fluid (CSF), white-matter (WM) and gray-matter (GM) was performed on the brain-extracted T1w using fast (FSL 6.0.5.1:57b01774, RRID:SCR_002823, Zhang, Brady, and Smith 2001). Brain surfaces were reconstructed using recon-all (FreeSurfer 6.0.1, RRID:SCR_001847, Dale, Fischl, and Sereno 1999), and the brain mask estimated previously was refined with a custom variation of the method to reconcile ANTs-derived and FreeSurferderived segmentations of the cortical gray-matter of Mindboggle (RRID:SCR_002438, Klein et al. 2017). Volume-based spatial normalization to two standard spaces (MNI152NLin2009cAsym, MNI152NLin6Asym) was performed through nonlinear registration with antsRegistration (ANTs 2.3.3), using brain-extracted versions of both T1w reference and the T1w template. The following templates were selected for spatial normalization: ICBM 152 Nonlinear Asymmetrical template version 2009c [Fonov et al. (2009), RRID:SCR_008796; TemplateFlow ID: MNI152NLin2009cAsym], FSL's MNI ICBM 152 non-linear 6th Generation Asymmetric Average Brain Stereotaxic Registration Model [Evans et al. (2012), RRID:SCR_002823; TemplateFlow ID: MNI152NLin6Asym].

Normalization template: ICBM 152 Nonlinear Asymmetrical template version 2009c [Fonov et al. (2009), RRID:SCR_008796; TemplateFlow ID: MNI152NLin2009cAsym]

Noise and artifact removal: To control for noise signals in the fMRI data, the first-level GLMs included framewise displacement, 24 head motion parameters (three translations and three rotations, their first and second-order derivatives) and 12 parameters for WM, CSF and global signal change (their first and second-order derivatives), obtained during preprocessing. As part of the additional parameters for denoising, 9 cosine regressors were modeled by Nilearn.

Volume censoring: No volume censoring was performed.

Statistical modeling & inference

Model type and settings: We used univariate (BOLD adaptation) and multivariate (RSA) analyses. We further used Generalized Psychophysiological Interaction (gPPI) analysis to examine changes in the functional connectivity. We first estimated effects (contrasts) for each participant as a first-level fixed-effects analysis and then used second-level group inferential statistics.

Effect(s) tested: Short description of analyses (see Methods for details):

RSA:
We used RSA to investigate a 6-fold grid-like modulation of bold signal for abstract directions between pairs of stimuli. We anticipated greater similarity between fMRI patterns for directions differing by multiples of 60 degree (with a remainder of 0 degree when dividing their angular difference by 60 degree) compared to directions whose angular difference results in a remainder of 30 degree.

BOLD adaptation analyses:
The BOLD response was expected to show different levels of adaptation depending on the abstract similarity between the pairs of stimuli. To test for the adaptation effect, each GLM included a parametric regressor, with the expected modulation aligned to the presentation time point and duration of the second stimulus of each pair.

gPPI analysis:
The analysis was implemented in the CONN toolbox. In the first-level model, we modeled two PPI regressors as a product of BOLD time series data from the seed ROI and two task conditions. To make a statistical inference on the difference in functional connectivity between the two task conditions (two levels of abstract similarity between pairs of stimuli) at the group level, we used the parameter estimates derived from the first-level model. Specifically, for each participant, we contrasted the whole-brain map of the interaction term between the two conditions [1,-1] within the toolbox and then used second-level group inferential statistics.

Specify type of analysis: ☐ Whole brain ☐ ROI-based ☒ Both

Anatomical location(s): The ROI masks were taken from the Julich Brain Atlas and Harvard-Oxford Brain Atlas. We resampled all masks to match the 2.5mm resolution of the functional images.

Statistic type for inference (See Eklund et al. 2016): Whole-brain analyses: voxel-wise.
ROI analyses: mean effect averaged over voxels of the ROI.

Correction: A false discovery rate (FDR) correction applied at a voxel-level threshold of $p < 0.01$

Models & analysis

n/a	Involved in the study
☐	☒ Functional and/or effective connectivity
☒	☐ Graph analysis
☒	☐ Multivariate modeling or predictive analysis

Functional and/or effective connectivity: Generalized psychophysiological interaction (gPPI) analysis: a functional connectivity analysis identifying the task-based change in correlation of BOLD time-series between the seed region and all other voxels or ROIs. The first-level model included a total of five regressors, where (i) two of these regressors represented the task conditions, serving as the two main psychological factors. (ii) Another regressor captured the BOLD time series data from the seed ROI, representing the main physiological factor. (iii) The remaining two regressors modeled the PPI term as the product of (i) and (ii), capturing the interaction term within the analysis.
